# Analysis of Paraben and Bisphenol A Exposure in Relation to Food Intake Levels and Health Risk Assessments in the Taiwanese Population

**DOI:** 10.1111/1750-3841.70652

**Published:** 2025-10-30

**Authors:** Kai‐Wei Liao, Hsuan‐Cheng Yen, Chun‐Huei Chang, Wen‐Harn Pan, Mei‐Lien Chen

**Affiliations:** ^1^ School of Food Safety, College of Nutrition Taipei Medical University Taipei Taiwan; ^2^ Institute of Environmental and Occupational Health Sciences, School of Medicine National Yang‐Ming Chiao Tung University Taipei Taiwan; ^3^ College of Public Health Taipei Medical University Taipei Taiwan

**Keywords:** bisphenol A, dietary intake, exposure assessment, health risk assessment, human biomonitoring, parabens

## Abstract

Parabens (PBs) and bisphenol A (BPA) are common endocrine‐disrupting chemicals commonly encountered in daily life, yet research on their associations with food groups remains limited. To address this, a nationwide study in Taiwan evaluated the exposure risks of PBs and BPA. This study measured urinary concentrations of three PBs—methyl‐paraben (MP), ethyl‐paraben (EP), and propyl‐paraben (PP)—together with BPA using ultra‐performance liquid chromatography coupled with quadrupole time‐of‐flight mass spectrometry (UPLC‐QToF‐MS), using data from 706 participants in the Nutrition and Health Survey in Taiwan (NAHSIT). The median creatinine‐adjusted concentrations for MP, EP, PP, and BPA were 17.54, 0.97, 1.60, and 1.19 µg/g cre., respectively. Multiple linear regression identified a positive linear association was identified between EP exposure and oil group consumption in the ≥ 65 years group (β = 0.194, *p* = 0.04). G‐computation analysis was applied to estimate the population‐average causal effects of food group consumption on PBs and BPA levels, revealing distinct dietary associations with both compounds. For PBs, significant positive correlations were observed with fruits (+9.1% for EP in the 19–64 years group, *p* = 0.036), snacks (+1.7% for MP [*p* = 0.023] to +3.8% for EP [*p* = 0.006] in the 12–18 years group), seasoning (+19.6% for MP in the 12–18 years group, *p* = 0.009), and oils (+55.6% for EP in the ≥ 65 years group, *p* = 0.025), while negative correlations were noted with livestock (−12.0% for MP in the 12–18 years group, *p* = 0.030) and other foods (−5.1% for PP in the 19–64 years group, *p* = 0.034). For BPA, positive correlations were identified with protein (+8.7% in the ≥ 65 years group, *p* = 0.026) and fish and seafood (+24.4% in the ≥ 65 years group, *p* = 0.049). A probabilistic health risk assessment was conducted using Monte Carlo simulation to account for variability and uncertainty in exposure estimates, providing a distribution of potential risks and identifying the likelihood of exceeding acceptable daily intakes. Following the probabilistic simulation, the results showed that all hazard indices for PB exposure were below one, indicating no significant risk. However, the hazard quotient of the estimated daily BPA intake exceeded the tolerable level established by EFSA (0.2 ng/kg bw/day) across all age groups, suggesting potential health risks. To our knowledge, this is the first nationwide study to investigate the associations of urinary PBs and BPA with food group consumption in the general population of Taiwan, highlighting its novelty and public health relevance.

## 1 Introduction

Endocrine‐disrupting chemicals (EDCs) are defined by the International Programme on Chemical Safety (IPCS) as exogenous substances or mixtures that alter the functions of the endocrine system and consequently cause adverse health effects in an intact organism, its progeny, or (sub) populations (WHO 2012). Many EDCs are found in nature, such as naturally occurring compounds such as phytoestrogens derived from plants, and in artificial products, such as pesticides; plastic components like bisphenol A (BPA) and phthalates; as well as food preservatives and personal care products (PCPs) such as parabens (PBs) (Schug et al. 2015).

Parabens (PBs) are a group of p‐hydroxybenzoic acid esters with various alkyl chains that result in different compounds, such as methyl‐paraben (MP), ethyl‐paraben (EP), propyl‐paraben (PP), and butyl‐paraben (BP). PBs are mainly used as preservatives in PCPs and foods due to their higher antimicrobial activity, lower water solubility, and increased oil solubility (Soni et al. 2002). Previous studies have indicated that different food items contain different varying levels of PBs; for example, higher PB concentrations have been documented in processed foods than in unprocessed foods (Wei et al. 2021). As a result of their widespread use in consumer products, PBs have become ubiquitous in daily life (Zhou et al. 2022). Humans are primarily exposed to PBs through the dermal route and daily ingestion. Such exposure may lead to adverse health effects, and epidemiological studies have provided evidence of a correlations between PB exposure and health outcomes, including altered endocrine activities, oxidative stress, immune modulation, and even breast cancer (Fransway et al. 2019; Hager et al. 2022; Huang et al. 2022; Nowak et al. 2018; Zhao et al. 2021).

BPA was first synthesized in 1891 and later used to produce polycarbonate and epoxy resins, and this monomer is part of many products, such as children's toys, plastic containers, and food packaging coatings, etc. (Geens et al. 2011; Ribeiro, Ladeira, and Viegas 2017; Shafei et al. 2018). Humans are primarily exposed to BPA through oral route (Vandenberg et al. 2007) With the global use of plastic materials and plasticizers, BPA has become ubiquitous in daily life, as evidenced by human biomonitoring studies reporting a detection frequency of BPA in urine exceeding 90% (Bushnik et al. 2010; Geens et al. 2014). Epidemiological studies have linked exposure to BPA to human oxidative stress and possible disruptions of endocrine signaling pathways (Murata and Kang 2018; Steffensen et al. 2020). Furthermore, associations have been founded between BPA exposure and conditions such as breast cancer, cardiovascular diseases, diabetes, and obesity (Gao and Wang 2014; Guo et al. 2023; Hwang et al. 2018; Michalowicz 2014; Stillwater et al. 2020).

The use of liquid chromatography–tandem mass spectrometry (LC‐MS/MS) and ultra‐performance liquid chromatography–quadrupole time‐of‐flight mass spectrometry (UPLC‐QToF‐MS) to analyze urinary PBs and BPA has been widely applied in epidemiological studies and human biomonitoring (Lee et al. 2013; Heralde et al. 2025; Chen et al. 2022), with PBs and BPA frequently detected in human samples (Berman et al. 2014; Calafat et al. 2010; Chang et al. 2017; Huang, Chen, Chou, et al. 2022; Murawski et al. 2021). Although numerous studies have examined the associations of PBs and BPA with health outcomes, few have conducted probabilistic health risk assessments for these exposures using nationally representative samples. To our knowledge, this is the first nationwide study to simultaneously evaluate urinary PBs and BPA, investigate their associations with food group consumption, and perform a probabilistic health risk assessment in the general population of Taiwan.

The objectives of the present study were therefore to: (1) determine urinary concentrations of PBs and BPA in the general population of Taiwan, (2) explore associations between PB and BPA exposure and 24‐h dietary intake recall data, and (3) perform a health risk assessment of PBs and BPA exposure in the general population of Taiwan.

## 2 Materials and Methods

### 2.1 Study Population

We separated the different age groups according to human developmental stages, as each group may have different health and risk factors. The age groups were defined based on both human developmental stages and the school or educational system in Taiwan. Participants and biospecimens were obtained from the Nutrition and Health Survey in Taiwan (NAHSIT), which was conducted using a multistage stratified cluster sampling design. To ensure adequate participation from the general population of Taiwan, the sample size for each age and gender group was determined based on the population density and urbanization level of each city, using a probability proportional to size (PPS) sampling approach. Additionally, pregnant or breastfeeding women, older audlts with dementia who had lost communication abilities, and residents under institutional care were excluded from this study. After applying these inclusion and exclusion criteria, participants who provided sufficient urine samples for analysis of PBs and BPA and completed a 24 h dietary recall questionnaire were included in this study. In total, 706 participants were enrolled in this cross‐sectional study, including 189 children aged 6–11 years from NAHSIT 2012, 230 adolescents aged 12–18 years from NAHSIT 2010 and 2011, and 137 adults aged 19–64 years and 150 elderly participants aged ≥ 65 years from NHAHSIT 2005–2008 (Pan et al. 2011; Tu et al. 2011). Different survey periods focused on different age groups. Detailed information on the sample collection methods has been described in previous studies (Liao et al. 2022; Lin et al. 2021; Pan et al. 1999; Tu et al. 2011), and the study flowchart of participant recruitment is presented in Figure S1 (Figure S1). All urine samples and questionnaire data in this study were collected simultaneously. The questionnaire contained basic sociodemographic data (e.g., age and gender), anthropometric data (e.g., weight and height), and daily food consumption weights derived from the 24 h dietary recall. The interviewers asked participants about the foods and dishes they had consumed during the previous 24 h and estimated the amounts consumed. The cooked food weights (g) were obtained from the 24 h dietary recall were subsequently converted into food‐cooked weights. In the current study, based on the six food groups defined by the Taiwan Daily Food Guide (Health Promotion Administration 2018), food items were further categorized into the following subgroups for analysis: grains (rice and their products, wheat and flour products, starchy roots and tubers and their products, seeds and their products); oils (vegetable oil, animal fat and oil, nuts and their products); poultry (chicken and their products, ducks and their products, other poultry and their products); livestock (pork and their products, beef and their products, other livestock and their products); fish and seafood (freshwater fishes, saltwater fishes, other fishes, viscera, and their products, other seafood and their products); protein (eggs and their products, dairy, soybeans and their products, special nutritional food); vegetables (dark green leafy vegetables, light green leafy vegetables, bamboo shoots, cucurbits, legumes, mushrooms, other vegetables and their products, pickled vegetables, marine vegetables, seasoned vegetables); fruits (fresh fruits, fruit products, fruit juice); snacks (bread, pastries and cookies, candy, ice products and sweetened beverages, processed fruit juice, other snacks); alcohol; seasonings (sugar, salt, soy sauce, other seasonings); and others (instant noodles, sandwiches and burgers, steamed buns and dumplings, soup, others). The detailed 24 h dietary recall data of the study population are presented in Table S1. The design and validation of the dietary nutrient intakes assessed by 24 h recall were detailed elsewhere (Pan et al. 1999). Initial void spot urine specimens were collected, aliquoted, and kept in dry ice during transportation to the laboratory. Subsequently, they were frozen at −80°C until analysis. The institutional review board of National Yang‐Ming Chiao Tung University approved this study (IRB no. YM110016E).

### 2.2 Measurement of Parabens and Bisphenol A in Urine

The pretreatment method was modified from Chen et al. (Chen et al. 2005). A urine sample (500 µL) was spiked with 25 µL of an internal standard solution and thoroughly vortexed for 1 min. Then 1 mL of 1 M ammonium acetate solution (pH = 5.3) was added to each urine sample to adjust the pH, and the sample was deconjugated using 20 µL of β‐glucuronidase. All samples were incubated at 37°C for 15 h in a shaking water bath. Before cleaning up with solid‐phase extraction (SPE), 300 µL of 1N hydrochloric acid was added to all samples to adjust the mixtures to pH 3, and samples were centrifuged at 3000 rpm for 5 min. The deconjugated urine samples were cleaned with SPE cartridges (Elute‐PH, 100 mg/mL, Agilent, USA). Initially, the SPE cartridges were preconditioned using 2 mL of methanol, followed by 1 mL of acidified deionized water at pH 3. Subsequently, we added the supernatant of a centrifuged urine sample into a cartridge and washed it with 1 mL of deionized water. Finally, analytes were eluted with 1 mL of methanol. All samples were filtered through a 0.25 µm polytetrafluoroethylene membrane and transferred to 2 mL autosampler glass vials for instrumental anaylsis.

In this study, PBs and BPA concentrations in urine samples were quantified and analyzed using UPLC‐QToF‐MS (XEVO G2‐XS Q‐Tof, Waters, USA). The analytical approach employed a high‐performance ACQUITY UPLC BEH C18 column with a particle size of 1.7 µm and dimensions of 2.1 × 100 mm (Waters, USA). The mobile phase consisted of a binary mixture of acetonitrile and deionized water. The column temperature was set at 40°C, and a constant flow rate of 0.4 mL/min was maintained throughout the analysis. A small injection volume of 3 µL was utilized, reflecting the sensitivity of the method. The electrospray ionization (ESI) source operated in negative ion mode, with a source temperature of 140°C. These instrumental parameters collectively facilitated the efficient separation and accurate quantification of PBs and BPA in urine samples, providing a robust analytical framework for investigating the presence and concentrations of these compounds in biological matrices. The retention times of MP, EP, PP, and BPA were 1.36, 2.10, 3.44, and 3.74 min, respectively. The MS/MS transitions selected for MP was m/z 151.04→92.03, that for EP was m/z 165.05→92.03, that for PP was m/z 179.07→92.03, and that for BPA was m/z 227.12→133.07. The internal standard transitions selected for ^13^C_6_‐MP m/z were 157.06→98.05, those for ^13^C_6_‐EP m/z were 171.08→98.05, those for ^13^C_6_‐PP m/z were 185.09→98.05, and thos for ^13^C_12_‐BPA m/z were 239.18→139.09.

Values of the limit of detection (LOD)/limit of quantification (LOQ) for MP, EP, PP, and BPA were 0.03/0.11, 0.02/0.06, 0.02/0.07, and 0.11/0.37 ng/mL, respectively. The linearity of MP, EP, PP, and BPA was determined from 0.5 to 25 ng/mL, and the regression coefficients for PBs and BPA were above 0.995. MP, EP, PP, and BPA recovery rates ranged from 90.82% to 112.81%, 85.00% to 91.35%, 87.97% to 93.67%, and 87.01% to 96.25%, respectively. Coefficients of variance were all below 18.34%.

Urinary creatinine (cre.) levels were determined using a commercial kit (Eagle Diagnostics, Desoto, TX, USA). In summary, 0.1 mL of urinary samples was combined with 3 mL of 3.3 mM picric acid and incubated at 37°C for 15 min in a shaker bath. Quantification was performed using a spectrophotometer at a wavelength of 510 nm.

### 2.3 Estimated Daily Intake of Parabens and Bisphenol A

In this study, we calculated the estimated daily intake (EDI) levels to estimate PBs and BPA exposure doses. PBs and BPA EDI levels were determined using Equation 1. Based on the model utilizing creatinine‐adjusted urinary concentrations, this equation was modified from previous studies (Wei et al. 2022):

(1) $$\mathrm{EDI}\;=\frac{\mathrm{UE}\;\times \;\mathrm{CE}}{F_{\mathrm{UE}}\times \;\mathrm{BW}}$$

where UE (µg/g cre.) is the urinary concentration of PBs and BPA adjusted by creatinine; creatinine excretion (CE, g/day), daily creatinine excretion rate normalized by body weight (BW, kg) and height (ht, cm); F_UE_, is the urinary excretion factor of the target compound (F_UE_ is 17.4% for MP, 13.7% for EP, 8.6% for PP, and 100% for BPA, respectively (Moos et al. 2016; Moos et al. 2017; Shin et al. 2019).

The age, BW, ht, and sex‐specific creatinine‐adjusted values for CE rates were provided (Mage et al. 2004; Mage et al. 2008) as follows:

Adults (≥ 18 years old)

$$\mathrm{CE}=1.93\times \left(140-\mathrm{Age}\right)\times BW^{1.5}\times ht^{0.5}\times 10^{-6}\cdots \left(\mathrm{for}\;\mathrm{males}\right)$$

$$\mathrm{CE}\;=1.64\times \left(140-\mathrm{Age}\right)\times BW^{1.5}\times ht^{0.5}\times 10^{-6}\cdots \left(\mathrm{for}\;\mathrm{females}\right)$$

Minors (< 18 years old)

$$\begin{array}{ccc} & & \mathrm{CE}\;=\;\mathrm{ht}\;\times \left\{6.265+0.0564\times \left(\mathrm{ht}\;-168\right)\right\}\;\times 10^{-3}\cdots \\ & & \left(\mathrm{for}\;\mathrm{males}\;\mathrm{with}\;\mathrm{ht}\;<168\;\mathrm{cm}\right) \end{array}$$

$$\begin{array}{ccc} & & \mathrm{CE}\;=\;\mathrm{ht}\;\times \left\{6.265+0.2550\times \left(\mathrm{ht}\;-168\right)\right\}\;\times 10^{-3}\cdots \\ & & \left(\mathrm{for}\;\mathrm{males}\;\mathrm{with}\;\mathrm{ht}\;\geq 168\;\mathrm{cm}\right) \end{array}$$

$$\begin{array}{ccc} & & \mathrm{CE}\;=2.045\times \mathrm{ht}\;\times \exp\left\{0.01552\times \left(\mathrm{ht}\;-90\right)\right\}\;\times 10^{-3}\cdot \\ & & \left(\mathrm{for}\;\mathrm{females}\right) \end{array}$$

### 2.4 Health Risk Assessment

The European Food Safety Authority (EFSA) proposed an acceptable daily intake (ADI) levels of 0–10 mg/kg‐BW/day as consumer risk assessments for MP and EP and their sodium salts (EFSA 2004). The European Medicines Agency (EMA) established a toxicological ADI of PP at a dose of 1.25 mg/kg‐BW/day (European Medicines Agency 2015). In April 2023, the EFSA released a revised safety assessment for BPA and established a tolerable daily intake (TDI) of 0.2 ng BPA/kg‐BW/day (EFSA Panel on Food Contact Materials et al. 2023).

The Hazard Quotient (HQ) for each compound and the hazard index (HI), defined as the sum of HQ values for all compounds, were calculated for risk characterization using Equations 2 and 3:

(2) $$\mathrm{HQ}\;=\frac{\mathrm{EDI}}{\mathrm{ADI}\;\left(\mathrm{or}\;\mathrm{TDI}\right)}$$

(3) HI=∑HQ

In this study, we calculated the HQ sum of MP, EP, and PP for the HI. A calculated HQ or HI greater than 1 indicates that a population exposed to this compound has a potential risk, while a value below 1 indicates an acceptable or negligible risk.

### 2.5 Probabilistic aRisk Assessment

Probabilistic risk assessment comprises a group of techniques that incorporate variability and uncertainty into the risk assessment process. In this study, a Monte Carlo simulation was employed to quantify the variability in EDI across different age groups, based on creatinine‐adjusted urinary concentrations of PBs and BPA (UE), daily creatinine excretion rates (CE), and body weight (BW). Urinary concentrations were assumed to follow a log‐normal distribution, while body weight was modeled using a normal distribution. We used Crystal Ball (Oracle, Redwood City, CA, USA) to conduct the uncertainty analysis. Based on raw UE, CE, and BW data, we utilized the Kolmogorov‐Smirnov test to assess the optimal distribution (e.g., normal, lognormal, etc.) that best fits the data. A Monte Carlo simulation with 10,000 iterations was performed to ensure the stability and robustness of the results.

### 2.6 Statistical Analysis

Statistical analyses were conducted using SPSS 26.0 (IBM Corp., Chicago, IL, USA) with a significance level set to *p* < 0.05. Descriptive statistics were applied to sociodemographic and lifestyle data, including age, gender, and body mass index (BMI). In addition, all urinary concentrations were adjusted with creatinine concentrations. If concentrations were below the LOD, we instead used a value of LOD/2 for subsequent analyses. Furthermore, the Kruskal‐Wallis test was used to determine whether there were any differences in urinary concentrations of PBs and BPA between different age groups. Spearman's correlation analysis was used to compare correlations of urinary PBs and BPA concentrations with 24 h dietary recall data. Then, significant food groups related to PBs and BPA were subjected to a multiple linear regression analysis. G‐computation regression analysis was applied to estimate the population‐average causal effects of food group consumption on PBs and BPA levels, while adjusting for covariates. This approach also allowed us to calculate the proportion of positive and negative partial effects of each food group, providing a more robust framework than conventional regression when multiple correlated dietary exposures were considered. The results were visualized as stacked bar charts, with positive and negative coefficients representing the direction and magnitude of the associations. Distributions of urinary PB and BPA concentrations across different age groups were plotted using GraphPad Prism (version 8.0.2, La Jolla, CA, USA). The G‐computation models, along with their visualizations, were performed using R software (version 4.4.1, The R Foundation for Statistical Computing, Vienna, Austria).

## 3 Results

### 3.1 Sociodemographic Characteristics and 24 h Dietary Recall Data of the Study Population

In total, 706 participants were enrolled in this study. The average age of participants was 31.27 ± 25.70 years, and the mean BMI was 21.74 ± 4.74 kg/m^2^, with 31.7% having a BMI ≥ 24 kg/m^2^. The study included a nearly equal number of males and females (Table 1). The study population was largely the same as in our previous study, with some differences in the subjects included (Liao et al. 2022).

**TABLE 1:** Demographic characteristics of the study population.

Study population (N = 706)	N	% / Mean ± SD
Age (years)	706	31.27 ± 25.70
BMI (kg/m^2^) ^a^	701	21.74 ± 4.74
< 24	479	68.3
≥ 24	222	31.7
Gender		
Male	352	49.86
Female	354	50.14

^a^ : Five missing data. BMI classification was based on the Health Promotion Administration, Ministry of Health and Welfare, Taiwan: 18.5 ≤ BMI < 24 kg/m^2^ = normal weight, ≥ 24 kg/m^2^ BMI = overweight or obese.

Study subjects were divided into four age groups: 6–11, 12–18, 19–64, and ≥ 65 years old. The 24 h dietary recall data of the studied population are presented in Table 2. In the 6–11 and ≥ 65 age groups, grains were the most consumed food group, with median intakes of 314.45 g and 562.38 g, respectively. For the 12–18 and 19–64 age groups, snacks represented the primary food group consumed, with median intakes of 528.93 g and 433.77 g, respectively. Conversely, in the 6–11 and ≥ 65 age groups, snacks were the second most consumed food group, with median intakes of 238.29 g and 284.43 g, respectively. Meanwhile, in the 12–18 and 19–64 age groups, grains were the second most consumed food group, with median intakes of 343.58 g and 412.24 g, respectively (Table 2).

**TABLE 2:** 24 h dietary recall data (cooked weight, g) of the study population.

	6–11 years n = 188 ^a^			12–18 years n = 228 ^b^			19–64 years n = 136 ^a^			≥ 65 years n = 150		
	Median	Mean	SD	Median	Mean	SD	Median	Mean	SD	Median	Mean	SD
Food groups												
Grain	314.45	354.46	206.11	343.58	409.20	278.35	412.24	483.75	307.45	562.38	645.64	381.85
Oil	12.28	17.82	19.68	14.74	19.64	20.76	13.88	22.24	26.73	12.30	14.84	20.95
Poultry	0.00	28.91	49.64	9.48	51.98	79.56	0.00	17.05	33.05	0.00	8.55	23.67
Livestock	45.88	69.85	79.43	61.52	82.27	81.70	56.95	76.72	84.12	39.02	60.29	78.07
Fish and seafood	11.31	33.21	49.56	16.79	41.92	60.56	30.84	62.41	90.53	12.14	33.68	53.24
Protein	177.11	231.57	230.50	154.23	218.67	230.88	98.19	196.90	262.53	45.83	110.18	160.44
Vegetable	132.22	174.52	152.83	145.75	180.47	136.81	227.34	293.97	249.36	276.56	325.66	264.44
Fruit	84.86	121.60	147.90	23.25	108.22	151.04	182.42	252.70	259.83	120.23	171.86	194.54
Snack	238.29	367.36	394.63	528.93	671.28	604.33	433.77	742.36	840.68	284.43	493.32	745.35
Alcohol	0.00	1.12	4.04	0.00	3.80	24.30	0.10	25.41	124.49	0.00	15.11	76.14
Seasoning	26.45	49.34	69.38	30.17	52.53	60.91	22.92	58.79	165.23	15.01	27.19	36.56
Others	230.03	312.48	328.33	252.98	326.78	334.42	239.22	332.43	414.47	196.29	276.54	532.21

^a^ : One missing data; ^b^: Two missing data.

### 3.2 Urinary PBs and BPA Concentrations and Associations With 24 h Dietary Recall Data

**FIGURE 1:**
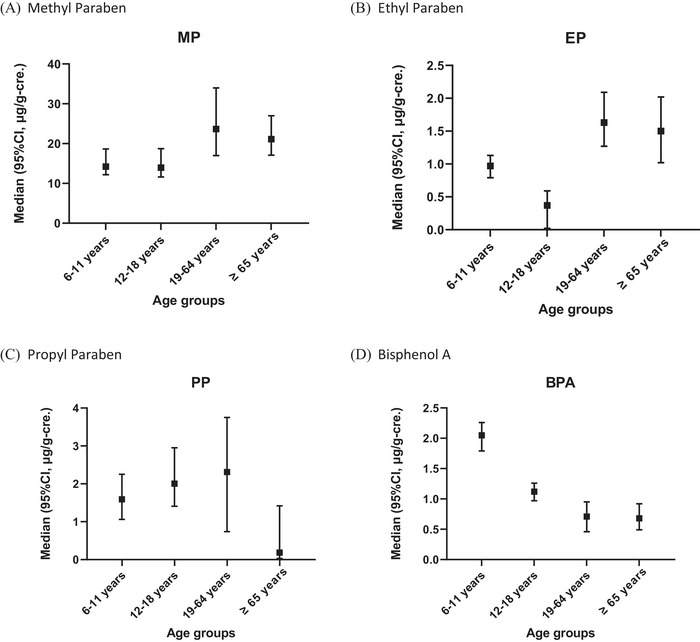
Distribution of urinary parabens and bisphenol A (µg/g‐creatinine) in different age groups.

Detection rates in the total population for MP, EP, PP, and BPA were 98.30%, 71.25%, 65.44%, and 80.74%, respectively. Median concentrations of MP/EP/PP/BPA in the 6–11, 12–18, 19–64, and ≥ 65 years groups were 14.23/0.97/1.59/2.05, 13.94/0.37/2.00/1.12, 23.64/1.63/2.31/0.71, and 21.10/1.50/0.18/0.68 µg/g‐cre., respectively (Figure 1, Table S2). Concentrations of creatinine‐unadjusted PBs and BPA are presented in Table S2.

Based on data from the 24 h dietary recall data (Table S1), the Spearman correlation of urinary PBs and BPA concentrations with 24 h dietary recall data was estimated. In the 6–11 years group, urinary EP, PP, and BPA concentrations were significantly positively correlated with the fish and seafood (r = 0.148, *p* < 0.05), seasoning (r = 0.151, *p* < 0.05), and alcohol (r = 0.155, *p* < 0.05) food groups, respectively. For the 12–18 years group, urinary EP concentrations were significantly negatively correlated with the oil (r = −0.182, *p* < 0.01), poultry (r = −0.176, *p* < 0.01), and livestock (r = −0.149, *p* < 0.05) food groups, and urinary PP concentrations were also significantly and negatively correlated with the grain (r = −0.174, *p* < 0.01), oil (r = −0.187, *p* < 0.01), alcohol (r = −0.132, *p* < 0.05), and seasoning (r = −0.150, *p* < 0.05) food groups. In the 19–64 years group, only urinary MP concentrations were significantly negatively correlated with the grain (r = −0.172, *p* < 0.05) food group. Among the ≥ 65 years group, urinary MP, EP, and PP concentrations were significantly positively correlated with the vegetable (r = 0.231, *p* < 0.01), seasoning (r = 0.205, *p* < 0.05), and fruit (r = 0.169, *p* < 0.05) food groups (Table 3).

**TABLE 3:** Spearman correlations coefficients of parabens and bisphenol A levels ^a^ (µg/g creatinine) with 24 h dietary recall data.

	6–11 years n = 188 ^a^				12–18 years n = 228 ^b^				19–64 years n = 136 ^a^				≥ 65 years n = 150			
Food group	MP	EP	PP	BPA	MP	EP	PP	BPA	MP	EP	PP	BPA	MP	EP	PP	BPA
Grain	0.018	0.015	−0.025	0.083	−0.119	−0.102	−0.174**	−0.068	−0.172*	−0.049	−0.159	0.092	0.072	−0.0004	0.024	−0.063
Oil	0.014	−0.045	0.018	0.119	−0.112	−0.182**	−0.187**	0.038	0.021	0.028	0.063	0.102	−0.010	0.100	0.032	0.010
Poultry	−0.051	−0.024	0.027	−0.035	−0.047	−0.176**	−0.099	−0.090	−0.126	−0.148	−0.079	0.136	0.024	−0.002	−0.095	−0.005
Livestock	0.134	−0.054	−0.097	0.021	−0.067	−0.149*	−0.098	−0.023	0.075	−0.026	0.012	−0.049	−0.031	−0.008	−0.111	0.007
Fish and seafood	0.035	0.148*	0.070	0.086	−0.121	−0.048	−0.082	0.018	0.067	0.128	0.100	0.006	−0.001	0.085	0.006	−0.048
Protein	−0.051	−0.056	0.019	−0.047	−0.082	0.084	−0.045	0.054	−0.097	−0.079	0.040	−0.018	−0.006	−0.044	0.092	0.059
Vegetable	0.062	0.051	−0.132	−0.002	−0.001	0.014	−0.118	0.028	0.091	0.112	0.004	0.091	0.231**	0.065	0.150	−0.089
Fruit	−0.068	0.013	−0.011	0.040	0.093	0.054	0.073	−0.039	0.029	−0.042	−0.076	0.049	0.159	0.077	0.169*	0.044
Snack	0.039	−0.074	0.043	0.064	0.033	−0.016	0.079	0.028	0.117	0.071	0.029	−0.061	0.009	−0.018	0.001	0.082
Alcohol	−0.075	0.123	0.092	0.155*	0.016	−0.094	−0.132*	0.117	0.065	−0.056	0.058	0.102	−0.020	0.160	−0.044	−0.080
Seasoning	0.117	0.102	0.151*	0.088	−0.029	−0.081	−0.150*	−0.065	0.111	0.114	0.118	−0.109	0.037	0.205*	0.093	0.006
Others	0.061	0.128	0.083	−0.032	−0.073	−0.022	−0.061	−0.048	0.056	−0.084	−0.109	0.052	0.029	0.098	−0.153	−0.154

^a^ : One missing data; ^c^: Two missing data. * *p*‐value < 0.05.

Table 4 presents relationships between urinary PBs and BPA concentrations and 24 h dietary recall data based on multiple linear regression analyses. Previous studies have indicated significant differences in PBs and BPA exposure characteristics between different sexes (Cheng et al. 2023; Gao et al. 2023). To ensure that dietary habits related to other food categories do not influence the association between the intake of the category under investigation and exposure, the analysis was adjusted for sex and other food groups in the multiple regression model. After adjusting for sex and other food groups, only urinary EP concentrations were significantly positively correlated with cooking oils consumed in the ≥ 65 years group (β = 0.194, 95% confidence interval (CI) = 0.009–0.378) (Table 4).

**TABLE 4:** Relationship between urinary parabens and bisphenol A concentrations and 24‐hour dietary recall data based on multiple linear regression analyses.

	6‐11 years (n= 189) ^a^			12‐18 years (n= 230) ^a^			19‐64 years (n= 137) ^a^			≥65 years (n= 150) ^a^		
	β	95% CI	*p*‐value	β	95% CI	*p*‐value	β	95% CI	*p*‐value	β	95% CI	*p*‐value
MP (µg/g creatinine)												
Constant	52.093			12.868			−7.068			42.869		
Grains	−0.084	−0.315 – 0.147	0.476	0.002	−0.024 – 0.028	0.885	−0.009	−0.115 – 0.098	0.875	0.003	−0.100 – 0.106	0.954
Vegetables	−0.035	−0.342 – 0.272	0.824	−0.008	−0.059 – 0.042	0.744	−0.060	−0.188 – 0.069	0.362	0.034	−0.114 – 0.183	0.649
R	0.095			0.123			0.218			0.045		
R^2^	0.009			0.015			0.048			0.002		
EP (µg/g creatinine)												
Constant	−2.181			−1.839			−13.678			−1.664		
Oils	−0.038	−0.198 – 0.121	0.638	−0.006	−0.086 – 0.074	0.881	−0.011	−0.173 – 0.152	0.898	**0.194**	**0.009 – 0.378**	0.040*
Poultry	0.038	−0.023 – 0.100	0.216	−0.016	−0.036 – 0.005	0.145	−0.079	−0.211 – 0.053	0.241	−0.058	−0.200 – 0.104	0.480
Livestock	0.002	−0.037 – 0.040	0.930	0.003	−0.018 – 0.024	0.792	0.018	−0.038 – 0.074	0.531	−0.014	−0.064 – 0.036	0.581
Fish and seafood	0.002	−0.059 – 0.062	0.952	−0.017	−0.044 – 0.011	0.228	0.005	−0.042 – 0.053	0.823	0.053	−0.022 – 0.129	0.164
Seasonings	−0.007	−0.052 – 0.037	0.743	0.022	−0.006 – 0.050	0.119	−0.005	−0.033 – 0.023	0.727	0.014	−0.094 – 0.122	0.800
R	0.135			0.200			0.333			0.234		
R^2^	0.018			0.040			0.111			0.055		
PP (µg/g creatinine)												
Constant	0.086			4.591			−13.209			5.748		
Grains	−0.006	−0.017 – 0.005	0.284	−0.003	−0.011 – 0.005	0.460	−0.005	−0.017 – 0.007	0.442	−0.003	−0.013 – 0.007	0.521
Oils	0.028	−0.086 – 0.143	0.625	0.001	−0.102 – 0.105	0.977	0.079	−0.062 – 0.219	0.271	−0.022	−0.204 – 0.161	0.816
Fruits	0.013	−0.002 – 0.027	0.089	−0.004	−0.018 – 0.009	0.533	0.008	−0.007 – 0.022	0.299	0.001	−0.018 – 0.020	0.918
Alcohol	0.023	−0.523 – 0.570	0.933	−0.029	−0.114 – 0.057	0.509	−0.003	−0.033 – 0.027	0.823	−0.018	−0.067 – 0.031	0.474
Seasonings	0.012	−0.020 – 0.044	0.475	−0.011	−0.045 – 0.024	0.552	0.016	−0.006 – 0.039	0.160	0.039	−0.063 – 0.141	0.449
R	0.222			0.164			0.386			0.135		
R^2^	0.049			0.027			0.149			0.018		
BPA (µg/g creatinine)												
Constant	2.292			0.784			1.452			3.400		
Alcohol	0.013	−0.121 – 0.147	0.847	0.003	−0.013 – 0.019	0.708	−0.001	−0.003 – 0.002	0.668	−0.004	−0.015 – 0.007	0.528
R	0.033			0.095			0.042			0.086		
R^2^	0.001			0.009			0.002			0.007		

a: Covariates including sex and other food groups. *p<0.05.

### 3.3 G‐Computation Analysis

**FIGURE 2:**
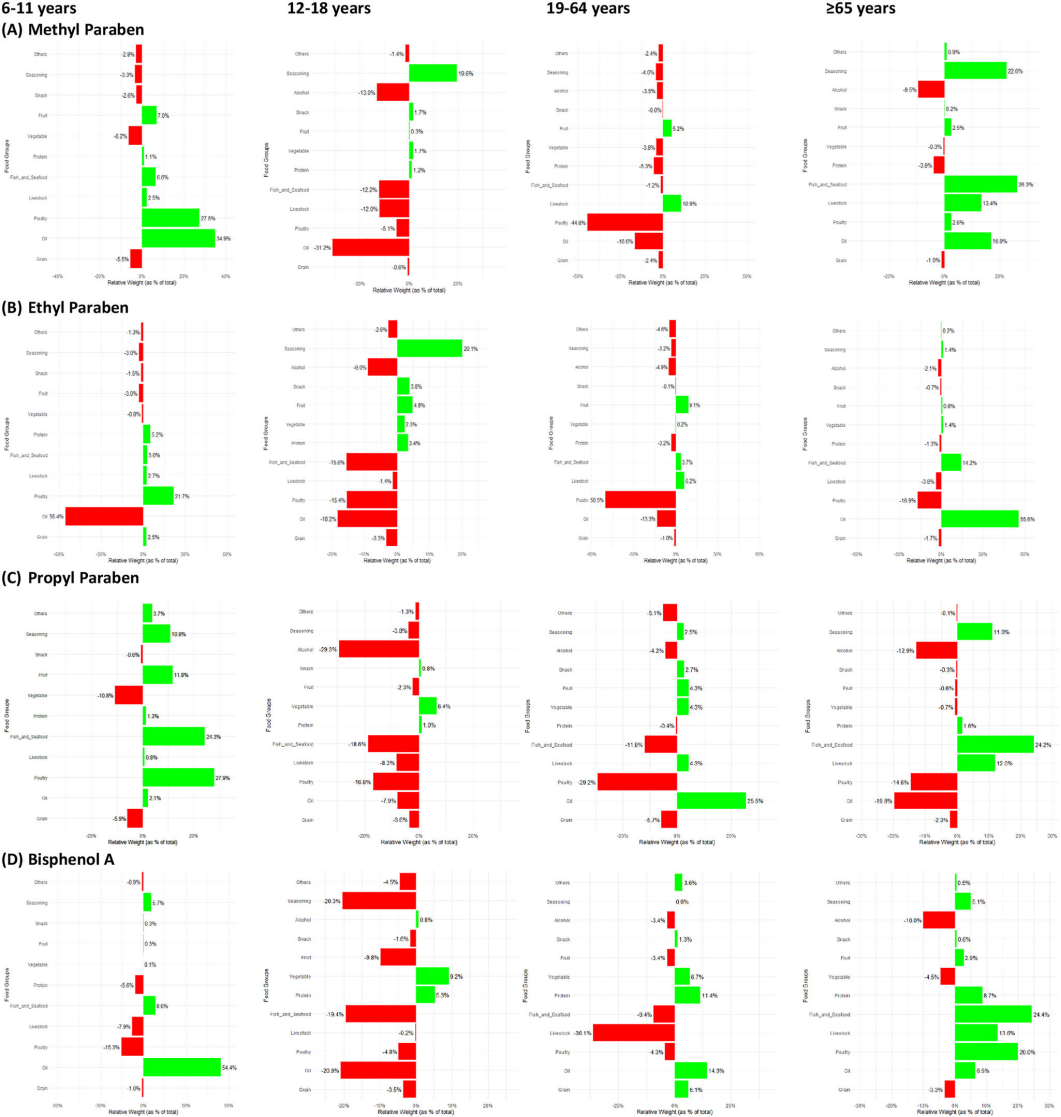
Weights represent the proportion of the positive or negative partial effect of Food Group Consumption on human exposure levels in the g‐computation models. Model‐adjusted sex.

Figure 2 shows the results of the g‐computation analysis stratified by age groups to investigate potential differences in these associations across demographic subgroups. The g‐computation model was adjusted for sex to account for potential confounding variables. For the 6–11 years group, there was a borderline positive association between PP exposure and fruit group consumption, with an estimated 11.8% increase in urinary concentrations associated with food group consumption (*p* = 0.062). In the 12–18 years group, MP exposure was significantly positively correlated with the consumption of seasoning (19.6%, *p* = 0.009) and snacks (1.7%, *p* = 0.023) groups, and negatively correlated with livestock group consumption (‐12.0%, *p* = 0.030). Additionally, EP exposure showed a significant positive association with snack group consumption (3.8%, *p* = 0.006). In the 19–64 years group, EP exposure was significantly positively correlated with fruit group consumption (9.1%, *p* = 0.036). PP exposure in this age group was also significantly positively correlated with snack group consumption (2.7%, *p* = 0.023) and negatively correlated with the consumption of other groups (−5.1%, *p* = 0.034). Among adults aged ≥ 65 years, EP exposure was significantly positively associated with oil group consumption (55.6%, *p* = 0.025). Furthermore, BPA exposure in this age group was found to be significantly positively correlated with the consumption of the protein group (8.7%, *p* = 0.026) and the fish and seafood group (24.4%, *p* = 0.049). These findings highlight age‐specific dietary intake associated with varying pollutant exposures across demographic groups (Figure 2).

### 3.4 Estimation of Daily PBs and BPA Intake Levels

We calculated EDI levels from urinary concentrations of PBs and BPA. Median values of MP/EP/PP EDIs were 1.53/0.13/0.32, 1.43/0.05/0.43, 2.46/0.20/0.48, 1.45/0.11/0.006 µg/kg‐BW/day for the 6–11, 12–18, 19–64, and ≥ 65 years groups, respectively. As to daily BPA intake levels, respective median values were 0.03, 0.02, 0.01, and 0.008 µg/kg‐BW/day for the 6–11, 12–18, 19–64, and ≥ 65 years groups. There were significant differences in intake levels of PBs and BPA among different age groups. The 19–64 years group had the highest median concentrations of urinary PBs, while the 6–11 years group had the highest median concentration of urinary BPA (Table 5).

**TABLE 5:** Distributions of estimated daily dietary parabens and bisphenol A intake (µg/kg‐BW/day) by different age groups.

	Group	n	Min	P25	P50	P75	Max	*p*‐value ^a^
**Methyl paraben**	6–11 years	189	0.002	0.55	1.53	3.42	355.2	0.002**
	12–18 years	229	0.001	0.58	1.43	4.02	47.9	
	19–64 years	136	0.003	1.03	2.46	6.02	147.8	
	≥ 65 years	147	0.001	0.56	1.45	3.36	142.7	
	Total	701	0.001	0.61	1.64	3.92	355.2	
**Ethyl paraben**	6–11 years	189	0.001	0.05	0.13	0.26	36.4	< 0.001**
	12–18 years	229	0.0003	0.001	0.05	0.23	16.3	
	19–64 years	136	0.0007	0.08	0.20	0.57	14.5	
	≥ 65 years	147	0.0006	0.02	0.11	0.33	8.52	
	Total	701	0.0003	0.003	0.12	0.30	36.45	
**Propyl paraben**	6–11 years	189	0.001	0.01	0.32	1.19	24.3	0.003**
	12–18 years	229	0.0004	0.004	0.43	1.75	31.9	
	19–64 years	136	0.0009	0.003	0.48	2.03	29.9	
	≥ 65 years	147	0.0005	0.002	0.006	1.21	23.7	
	Total	701	0.0004	0.003	0.33	1.57	31.9	
**Bisphenol A**	6–11 years	189	0.001	0.02	0.03	0.05	0.64	< 0.001**
	12–18 years	229	0.0003	0.01	0.02	0.03	0.69	
	19–64 years	136	0.0005	0.002	0.01	0.03	0.34	
	≥ 65 years	147	0.0005	0.002	0.008	0.02	0.64	
	Total	701	0.0003	0.007	0.02	0.04	0.69	

^a^ Comparison of different age groups by Kruskal‐Wallis test. **p < 0.01.

### 3.5 Health Risk Assessment of Daily Parabens and BPA Intake Levels

**FIGURE 3:**
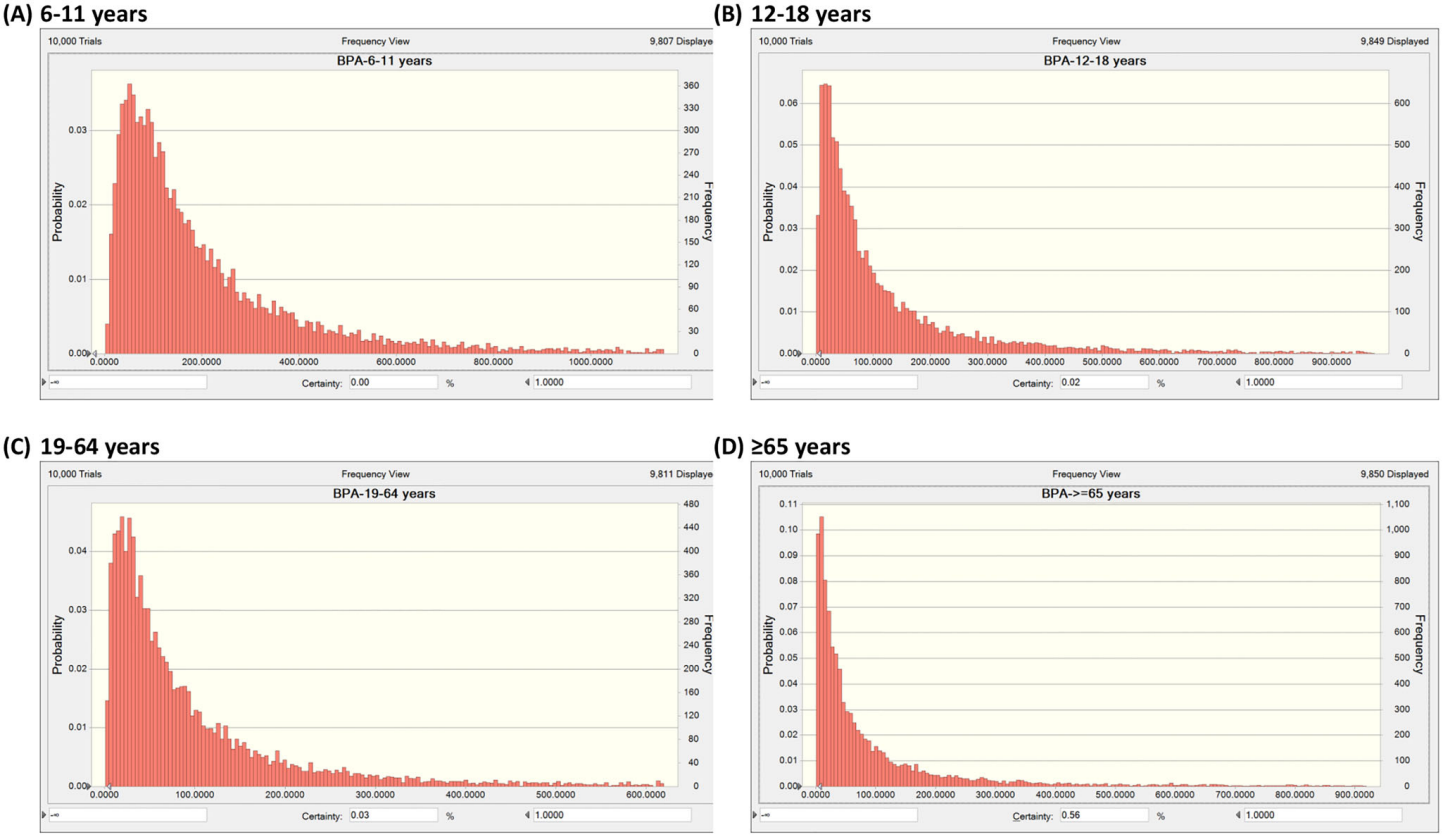
Probabilistic hazard quotients (HQs) of bisphenol A.

**FIGURE 4:**
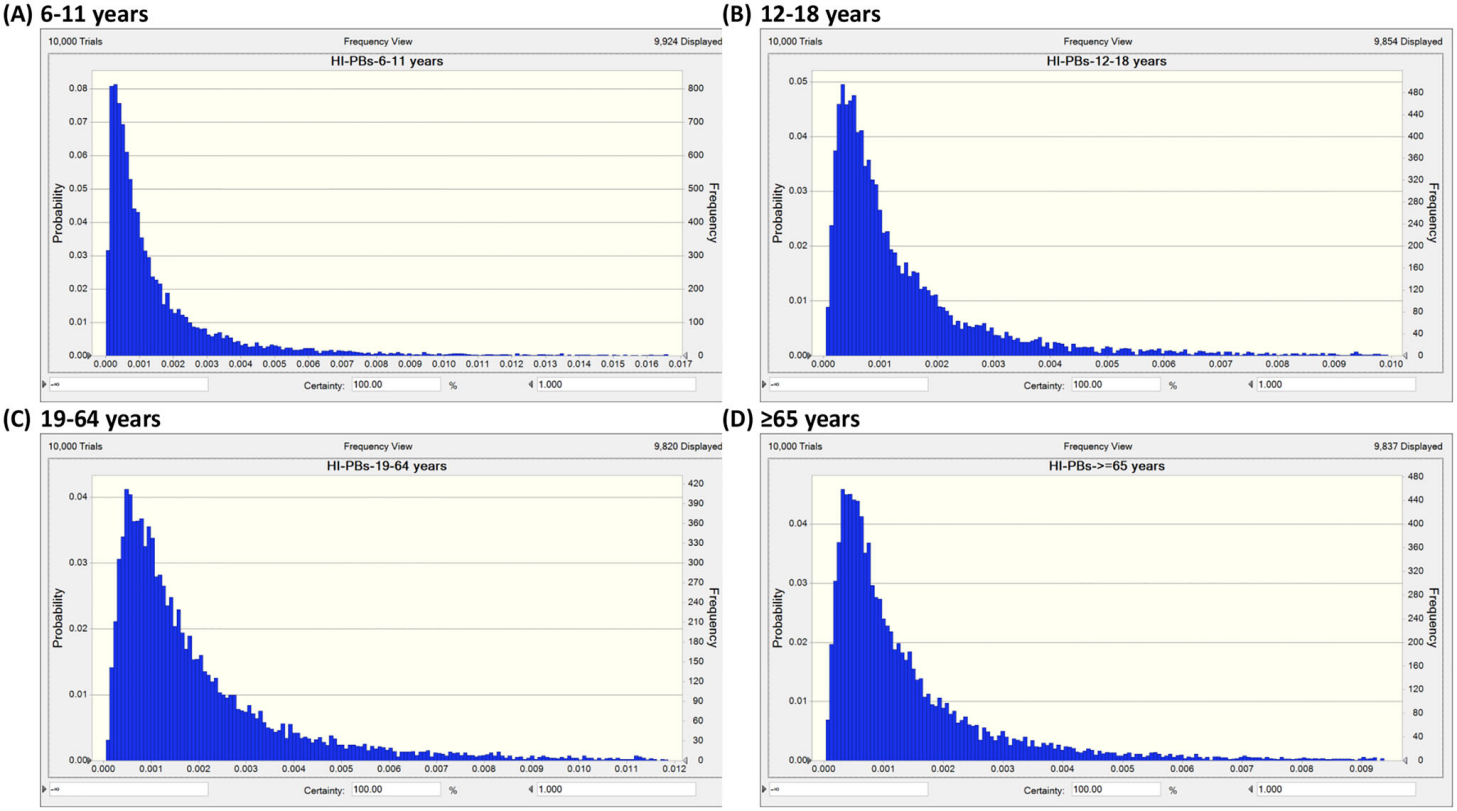
Probabilistic hazard index (HI) of parabens.

We utilized the EFSA proposed ADI values for MP and EP at 10 mg/kg‐BW/day, and for BPA at 0.2 ng/kg‐BW/day, as reference doses. Additionally, we employed the ADI established by the EMA for PP at a dose of 1.25 mg/kg‐BW/day as a reference dose. The HQs for MP, EP, PP, and BPA were calculated, and detailed distributions of HQs in different age groups are presented in Table S3. Following comparisons to reference doses, a probabilistic approach was employed to estimate distributions of PBs HIs (the summation of MP, EP, and PP) and BPA HQs. All HQ values of BPA in different age groups were observed to be ≥ 1, indicating potential health risks (Figure 3). Conversely, after calculating the HIs of PBs, it was observed that the HIs of PBs in different age groups were all < 1, suggesting no health risks (Figure 4).

## 4 Discussion

The study represent the large‐scale biomonitoring investigation of PBs and BPA in Taiwan. After adjusting urinary concentrations by creatinine, concentrations of MP, EP, and PP in the 19–64 years group were the highest among all age groups. In contrast, BPA concentrations were highest among children aged 6–11 years. After adjusting for covariates, EP concentrations were positively associated with the intake of the cooking oil food group in the ≥ 65 years group, whereas no food groups showed a statistically significant correlation with BPA exposure. G‐computation analyses provided additional insights into relationships between dietary intake and PB and BPA exposure. After the probabilistic simulation analysis, the sum of the EDI of PBs in each age group did not exceed reference doses. Conversely, the estimated BPA daily intake distribution in each age group showed that 100% exceeded the TDI of 0.2 ng/kg BW/day published by the EFSA. The exposure risks for BPA identified in this study are consistent with a report from the EFSA, indicating that consumers in all age groups, both with average and high exposure to BPA, exceeded the TDI.

Many studies have reported a high detection rate (> 90%) of PBs (Calafat et al. 2010; Huang, Chen, Chou, et al. 2022; Kang et al. 2016; Murawski et al. 2021; Quiros‐Alcala et al. 2018), and the present study also exhibited high detection rates (50%–98.7%). Urine is a major elimination pathway for xenobiotics, including EDCs, as these compounds undergo glucuronidation to increase polarity and facilitate excretion (Johnson et al. 2012; Perreault et al. 2013). Therefore, urinary concentrations are widely recognized as reliable biomarkers of human exposure. In a previous study in Taiwan, urinary concentrations of MP, EP, and PP were examined in adults (≥ 18 years old, n = 271) and minors (< 18 years old, n = 95) (Huang, Chen, Chou, et al. 2022). That study found significantly higher median concentrations of MP, EP, and PP in adults (MP: 397 ng/mL, EP: 38.8 ng/mL, PP: 117 ng/mL) than in minors (MP: 149 ng/mL, EP: 13.3 ng/mL, PP: 57.4 ng/mL, all *p* < 0.001). Furthermore, the median concentrations of PBs in the general population were in the order of MP > PP > EP (Huang, Chen, Chou, et al. 2022). The current study yielded consistent findings, showing that the median concentrations of urinary MP (23.64 ng/mL), EP (1.63 ng/mL), and PP (2.31 ng/mL) in the 19–64 years age group were higher than in other age groups, with MP showing the highest concentration followed by PP and EP. However, the concentrations observed in our study were several‐fold lower, which may be directly related to differences PCPs use among populations. In a Korean study, adults (aged 19–69 years) showed the highest median concentrations of MP (254 ng/mL), EP (53.4 ng/mL), and PP (24.0 ng/mL) compared to adolescents and children (Kang et al. 2016). Compared with data from the US National Health and Nutrition Examination Survey (NHNAES) studies (with median concentrations of MP of 50.1–58.8 µg/g‐cre. and PP of 5.2–8.27 µg/g‐cre.), concentrations found in the current study (MP: 17.54 µg/g‐cre.; PP: 1.76 µg/g‐cre.) were lower. Conversely, the median urinary EP concentrations were lower in the NHANES 2005–2006 study (< LOD, 1.0 µg/L) (Calafat et al. 2010), but higher in the NHANES 2007–2014 study (1.6 µg/L) (Quiros‐Alcala et al. 2018), compared with the current study (1.06 µg/L). When compared to the German Environmental Survey (GerES V) 2014–2017, participants aged ≦ 18 years in the current study exhibited higher median urinary concentrations of PBs than those in Germany (MP: 4.47 µg/g‐cre.; EP: 0.64 µg/g‐cre.; PP: < LOQ). Observed disparities in PB exposure levels in the previous Taiwanese study were attributed to differences in PCP use, while research conducted in Korea linked them to the BMI (Huang, Chen, Chou, et al. 2022; Kang et al. 2016) (Table S4). Overall, variations in urinary PBs and BPA concentrations across countries may reflect differences in the frequency and type of PCP use (Mok et al. 2025), as well as dietary patterns such as the consumption of packaged or processed foods (Monteagudo et al. 2021; Garcia‐Corcoles et al. 2018), all of which can contribute to disparities in exposure levels. At the same time, regulatory actions—such as restrictions on BPA in food contact materials (European Commission 2024)—vary widely between countries, and populations living under stricter regulatory environments may exhibit lower exposures.

The current study investigated the correlations between PBs and BPA exposure and dietary intake data obtained from 24 h recall. Based on the biological half‐lives of PBs (7.7 to 10.5 h) and BPA (8.5 to 24 h) (Nguyen et al. 2024; Stahlhut et al. 2009), 24 h dietary recall data are suitable for assessing correlations between intake levels and exposure. In addition, previous studies indicated that 24 h dietary recall surveys can yield good dietary assessment results (Shim et al. 2014; Strassburg et al. 2019). After accounting for other dietary categories, no significant linear correlation was observed between BPA and intake of any food groups. However, a significant positive correlation was found between the consumption of the oils used for cooking and nuts and seeds and urinary EP concentrations in the ≥ 65 age group. A plausible explanation is that EP, due to its lipophilic nature, may migrate more readily into fatty foods, thereby increasing exposure among individuals with higher oil intake. This interpretation is supported by previous studies reporting relatively higher EP concentrations in cooking oils compared with other food categories, and one study also identified canned tuna oil as having the highest EP levels among tested samples (Liao et al. 2013; Galvez‐Ontiveros et al. 2021). These findings suggest that both the fat content of foods and packaging‐related factors may contribute to the observed associations.

In 2004, the EFSA Panel established a group ADI of 0–10 mg/kg‐bw/day for the combined intake of MP and EP and their sodium salts, based on No‐Observed‐Adverse‐Effect Levels (NOAELs) of 1000 mg/kg‐bw/day for each compound in long‐term toxicity studies and in studies on sex hormones and male reproductive organs in juvenile rats. However, PP was excluded from this group ADI, as evidence indicated that, unlike MP and EP, it exerted effects on sex hormones and male reproductive organs in juvenile rats (EFSA 2004). The EMA established the ADI for PP at 1.25 mg/kg‐bw/day because the most appropriate No Observed Effect Level (NOEL) for deriving an ADI was 250 mg/kg‐bw/day, based on effects on the female rat reproductive system observed in a 19‐day study (European Medicines Agency 2015). For BPA, the mammary gland, prostate, and uterus were identified as the primary target organs of exposure, and EFSA established a temporary TDI of 4 µg/kg‐bw/day in 2015 (EFSA Panel on Food Contact Materials et al. 2023). However, in 2023, the EFSA Panel on Food Contact Materials, Enzymes and Processing Aids, after considering numerous studies and applying uncertainty factors, established a new TDI of 0.2 ng/kg‐bw/day to address potential health concerns associated with dietary BPA exposure in all age groups of the general population (EFSA Panel on Food Contact Materials et al. 2023). After estimating daily intake levels of PBs and BPA in different age groups, it was found that MP and EP had the highest median daily intake levels in the 19–64 years group, while PP was highest in the 12–18 years group. Notably, PP intake in adolescents was 45‐fold higher than that in the ≥ 65 years group. As for BPA, the highest daily dietary intake was observed in the 6–11 years group. The 6–11 years group's daily dietary BPA intake was even five times higher than that of the ≥ 65 years group (Table 5). Subsequently, we conducted a Monte Carlo simulation with 10,000 iterations in this study to estimate the distribution of the HQ of BPA and HIs of PBs in the general Taiwanese population. It was observed that BPA exposure exceeded the TDI of 0.2 ng/kg‐bw/day established by EFSA in 100%, 99.8%, 99.7%, and 94.4% of participants in the 6–11, 12–18, 19–64, and ≥ 65 years groups, respectively. Conversely, even after summing the EDIs of MP, EP, and PP and conducting the simulation, 100% of participants exhibited EDI levels below their respective reference doses. These findings suggest that adverse effects related to sex hormones or reproductive system are unlikely to be of concern for PBs in this population. The study presents the HQ of BPA in each age group, revealing that even in low‐exposure groups (percentile 5), the HQ in all age groups was ≥ 1, indicating potential risk. This result implies that BPA exposure among the Taiwanese population may contribute to endocrine‐disrupting effects, particularly adverse impacts on the mammary gland, prostate, and uterus. The finding is consistent with a report from the EFSA, where consumers with both average and high exposure to BPA across all age groups exceeded the TDI, indicating potential health concerns (EFSA Panel on Food Contact Materials et al. 2023). Regarding PBs, these results suggest that exposure to MP and EP poses a lower risk for the general population in Taiwan, regardless of age group.

In this study, g‐computation regression analysis was applied to evaluate the correlations between food group consumption and exposure biomarkers (MP, EP, PP, and BPA) across age groups, thereby highlighting distinct dietary influences. Oils consistently showed both strong positive and negative correlations, particularly with MP and BPA. Poultry exhibited prominent positive associations with EP and PP, while also appearing as a frequent negative correlate. Vegetables and seasonings demonstrated varying effects across age groups, with vegetables often associated with negative correlations. These discrepant results may be partly explained by differences in dietary behaviors across demographic subgroups, as consumption patterns of specific food groups vary by age. Previous studies have also reported that diet is an important source of PBs exposure, and that the magnitude of dietary contributions may differ by sex and age (Lee et al. 2025; Lim 2020). By using g‐computation, our study was able to estimate the contribution of different food groups to internal PBs exposure in different age groups, providing further insight into how dietary habits may differentially influence exposure in specific populations. These findings also help identify dietary patterns associated with higher exposure, offering opportunities to reduce exposure levels and ultimately mitigate potential health risks.

PBs are commonly used as preservatives in foods and cosmetics (Chatterjee et al. 2024). A previous study in Spain indicated that eggs, canned tuna, and bakery and baked products were the main contributors to dietary exposure to MP, EP, and PP in adolescent boys, while for adolescent girls, apples, pears, canned tuna, bakery, and baked products played a significant role (Monteagudo et al. 2021). Although the primary dietary source of PBs is not fully clear (Liao et al. 2013; Liao, Liu, and Kannan 2013; Maher et al. 2020), it can be explained by the presence of higher concentrations of antimicrobial agents in processed foods compared to unprocessed or raw foods (Soni et al. 2005). A previous study in China surveyed 13 food groups, revealing the highest mean concentration of EP in different food groups: condiments (42.8 ng/g), followed by cereals (5.39 ng/g), bean products (4.36 ng/g), fruits (6.89 ng/g), vegetables (10.9 ng/g), and cooking oils (4.22 ng/g), among others, and these results indicated that the consumption of oils has contributed to high exposure EP concentrations. The current study did not survey PB concentrations in the various food groups. However, we found that urinary EP concentrations were significantly positively correlated with the oil food group in the ≥ 65 years group. Although the group aged ≥ 65 years exhibited the lowest consumption of the oil food group (mean: 14.84 g) compared to the other age groups, subsequent consideration of EP exposure from other food groups revealed a correlation between oil intake and EP exposure.

BPA is widely used to produce polycarbonate (PC) and epoxy resins, derivatives of which are mainly used as food contact materials (Geens et al. 2011). In a previous study conducted in Taiwan, BPA was detected in 11 categories of 278 food samples collected from Taiwanese food markets and supermarkets, revealing the highest concentrations in canned foods, beverages, and oils, with significantly higher levels observed in high‐fat foods compared to low‐fat foods (Chang et al. 2019). Furthermore, the incomplete polymerization process may cause residues of BPA monomers in PC containers, and the coating can migrate into food, particularly during storage and processing at elevated temperatures (Kang et al. 2006). The EFSA also reported that BPA concentrations were significantly higher in packaged products (18.68 µg/kg) than in unpackaged products (1.5 µg/kg) (EFSA 2015). A previous study indicated that ingestion represents the primary route of exposure to BPA (Siddique et al. 2021). However, we found no correlations between food groups and urinary BPA concentrations. Detection rates of urinary BPA concentrations vary significantly across different countries' studies (15%–100%)(Berman et al. 2014; Chang et al. 2017; He et al. 2009; Park et al. 2016; Pirard et al. 2012), and detection rates and urinary BPA concentrations decrease as age increases. In the current study, we found that the 6–11 years group had the highest detection rate (94.1%) and median concentration of BPA (2.05 µg/g‐cre.). These results are consistent with a study in Belgium, where the detection rate in the < 20 years group was 100%, and the 0–6 years group had the highest median urinary BPA concentration (3.70 µg/g‐cre.) (Pirard et al. 2012). NHANES data in the US showed that the concentration of BPA in the urine of individuals in the 20 year old group was lower than those of < 20 years groups (CDC 2018) (Table S5). In Taiwan, the Taiwan Food and Drug Administration (TFDA) announced in 2013 that infant feeding bottles should not contain bisphenol A, and that the migration limit for polycarbonate food utensils, containers, and packaging should be set below 0.6 ppm (TFDA 2013). However, since the recruitment of participants in the 6–11 years group for this study took place in 2012, before the implementation of these regulations, their realtively higher BPA exposure levels may reflect pre‐regulatory conditions.

The strengths of this study include its large sample size, which represented the general Taiwanese population, and the age of the population, ranging from children to the elderly. This study provided comprehensive urinary biomonitoring data for PBs and BPA and examines their associations with dietary intake based on 24 h recall data. Furthermore, biomonitoring data were integrated with probabilistic health risk assesment to evaluate exposure levels and potential risks of PBs and BPA in the general population. These findings offer an importnat reference for the government in formulating management strategies to address exposure of the general population to PBs and BPA.

Although this study has contributed to our understanding of the topic, it has several limitations that should be acknowledged. This is a cross‐sectional study; therefore, causal relationships cannot be inferred . This study collected a single‐spot urine sample, which may not accurately reflect long‐term exposure levels of the general population to PBs and BPA. Daily changes in urinary PBs and BPA concentrations can lead to bias in the results. Moreover, the participants were asked to provide information for the past 24 h; however, the recall may elicit recall bias and measurement error from day‐to‐day variation. In addition, PBs and BPA are rapidly excreted into the urine because the half‐life of their metabolites is less than 24 h. However, previous studies have demonstrated that the half‐life of BPA is longer than previously expected (Stahlhut et al. 2009). Chronic low‐level exposure may lead to consistently elevated internal exposure levels. This study utilized a 24 h dietary recall method, which enabled a more accurate exploration of the relationship between diet and exposure. Furthermore, the recall method may not have captured complete and accurate exposure information about the subjects. For instance, the method did not investigate the materials of food containers, as materials in the containers can affect BPA exposure levels. As to non‐dietary factors, there was no questionnaire collecting the frequency of PCP usage, as PCP exposure is one of the main routes of exposure to PBs. The absence of these data restricts our ability to adjust for these factors and may weaken causal inference.

## 5 Conclusion

The study revealed significant associations between paraben and bisphenol A exposure and food intake, as well as the distribution of estimated daily exposure in the general population of Taiwan. As a result, exposure to bisphenol A poses potential health risks, underscoring the urgent need for strengthened monitoring and stricter regulatory control. In Taiwan, the use of bisphenol A‐containing plastic materials is prohibited in infant feeding bottles, the for polycarbonate food utensils, containers, and packaging, the maximum allowable bisphenol A migration limit is currently set at 0.6 ppm under specified test conditions. However, our findings indicate that bisphenol A exposure levels across all age groups exceed the tolerable daily intake, raising concerns that existing regulatory limits may not fully protect public health. As direct evidence from source tracking or food sampling is lacking, future research should focus on generating quantitative data by measuring concentrations of parabens and bisphenol A in various food groups and packaging materials. Such data would allow for more accurate exposure assessments, facilitate the validation of estimated intake values, and support improved risk evaluation and regulatory decision‐making.

## Author Contributions

**Kai‐Wei Liao**: writing–original draft, validation, writing–review and editing, formal analysis, data curation. **Hsuan‐Cheng Yen**: writing–original draft, methodology, formal analysis, data curation, investigation. **Chun‐Huei Chang**: investigation, methodology, formal analysis. **Wen‐Harn Pan**: conceptualization, funding acquisition, writing–original draft, writing–review and editing, methodology, project administration, supervision. **Mei‐Lien Chen**: conceptualization, funding acquisition, writing–original draft, writing–review and editing, validation, methodology, project administration, data curation, supervision, resources.

## Conflicts of Interest

The authors declare no conflicts of interest.

## Supporting information



Supplementary Tables: jfds70652‐sup‐0001‐Tables.docx
