# Supplementary material for: Analysis of Paraben and Bisphenol A Exposure in Relation to Food Intake Levels and Health Risk Assessments in the Taiwanese Population
Source: J Food Sci. 2025 Oct 30;90(11):e70652. doi: 10.1111/1750-3841.70652 (PMC12573222; doi:10.1111/1750-3841.70652)
Supplement: Supplementary file 1 — Supplementary Tables: jfds70652‐sup‐0001‐Tables.docx [file JFDS-90-0-s001.docx]

**Table S1.** Distribution of urinary parabens and bisphenol A levels (μg/g creatinine) in different age groups

|  | groups | Detection rate (%) | GM | GSD | min | P25 | P50 | P75 | Max | p-value |
| --- | --- | --- | --- | --- | --- | --- | --- | --- | --- | --- |
| MP | total | 98.30 | 15.69 | 5.36 | <LOD | 6.98 | 17.54 | 43.78 | 2990.26 | 0.003** |
|  | 6-11 years | 98.41 | 13.69 | 5.25 | <LOD | 5.89 | 14.23 | 32.95 | 2990.26 |  |
|  | 12-18 years | 98.70 | 12.43 | 4.92 | <LOD | 4.95 | 13.94 | 35.30 | 432.64 |  |
|  | 19-64 years | 97.10 | 22.32 | 5.92 | <LOD | 9.35 | 23.64 | 58.00 | 1524.31 |  |
|  | ≥ 65 years | 98.00 | 19.26 | 5.37 | <LOD | 8.56 | 21.10 | 55.17 | 2170.89 |  |
| EP | total | 71.25 | 0.45 | 13.68 | <LOD | <LOD | 0.97 | 2.44 | 273.86 | <0.001** |
|  | 6-11 years | 77.78 | 0.53 | 8.69 | <LOD | 0.37 | 0.97 | 1.98 | 273.86 |  |
|  | 12-18 years | 54.35 | 0.15 | 17.89 | <LOD | <LOD | 0.37 | 1.57 | 131.04 |  |
|  | 19-64 years | 85.51 | 1.29 | 9.40 | <LOD | 0.61 | 1.63 | 3.81 | 152.95 |  |
|  | ≥ 65 years | 75.33 | 0.78 | 11.27 | <LOD | 0.22 | 1.50 | 3.61 | 266.05 |  |
| PP | total | 65.44 | 0.62 | 22.39 | <LOD | <LOD | 1.60 | 8.43 | 182.72 | 0.083 |
|  | 6-11 years | 72.49 | 0.77 | 16.06 | <LOD | <LOD | 1.59 | 5.53 | 100.96 |  |
|  | 12-18 years | 70.87 | 0.73 | 22.19 | <LOD | <LOD | 2.00 | 8.53 | 126.71 |  |
|  | 19-64 years | 63.04 | 0.68 | 26.13 | <LOD | <LOD | 2.31 | 12.05 | 182.72 |  |
|  | ≥ 65 years | 50.00 | 0.35 | 27.90 | <LOD | <LOD | 0.18 | 9.28 | 153.07 |  |
| BPA | total | 80.74 | 0.88 | 3.99 | <LOD | 0.45 | 1.19 | 2.28 | 43.22 | <0.001** |
|  | 6-11 years | 94.18 | 1.75 | 2.71 | <LOD | 1.22 | 2.05 | 2.80 | 37.66 |  |
|  | 12-18 years | 87.39 | 0.84 | 3.58 | <LOD | 0.48 | 1.12 | 1.82 | 33.41 |  |
|  | 19-64 years | 67.39 | 0.53 | 4.30 | <LOD | <LOD | 0.71 | 1.61 | 18.39 |  |
|  | ≥ 65 years | 65.33 | 0.63 | 4.72 | <LOD | <LOD | 0.68 | 1.87 | 43.22 |  |

GM, Geometric mean; GSD, Geometric standard deviation; SD, Standard deviation; P25, P50, P75, percentile;

LOD, Limit of detection; LOD for MP was 0.03 ng/mL, EP and PP were 0.02 ng/mL, and BPA was 0.11 ng/mL.**p<0.01.

**Table S2.** Distribution of urinary parabens and bisphenol A levels (ng/mL) in different age groups

|  | groups | Detection rate (%) | GM | GSD | min | P25 | P50 | P75 | Max | p-value |
| --- | --- | --- | --- | --- | --- | --- | --- | --- | --- | --- |
| MP | total | 98.30 | 14.86 | 5.23 | <LOD | 6.33 | 16.91 | 34.87 | 1480.18 | 0.040* |
|  | 6-11 years | 98.41 | 12.59 | 4.97 | <LOD | 5.53 | 13.98 | 28.77 | 1480.18 |  |
|  | 12-18 years | 98.70 | 16.21 | 5.10 | <LOD | 6.22 | 19.32 | 44.91 | 754.09 |  |
|  | 19-64 years | 97.10 | 19.70 | 6.19 | <LOD | 8.08 | 20.97 | 44.89 | 1102.96 |  |
|  | ≥ 65 years | 98.00 | 12.40 | 4.78 | <LOD | 6.88 | 14.26 | 28.10 | 1290.03 |  |
| EP | total | 71.25 | 0.43 | 13.34 | <LOD | <LOD | 1.06 | 2.09 | 263.73 | <0.001** |
|  | 6-11 years | 77.78 | 0.49 | 9.42 | <LOD | 0.52 | 1.06 | 1.57 | 263.73 |  |
|  | 12-18 years | 54.35 | 0.19 | 17.88 | <LOD | <LOD | 0.74 | 2.25 | 118.59 |  |
|  | 19-64 years | 85.51 | 1.14 | 9.70 | <LOD | 0.75 | 1.31 | 3.34 | 179.86 |  |
|  | ≥ 65 years | 75.33 | 0.50 | 11.65 | <LOD | 0.06 | 1.02 | 2.07 | 141.00 |  |
| PP | total | 65.44 | 0.59 | 23.58 | <LOD | <LOD | 1.76 | 7.32 | 136.85 | <0.001** |
|  | 6-11 years | 72.49 | 0.70 | 16.87 | <LOD | <LOD | 1.69 | 4.84 | 84.35 |  |
|  | 12-18 years | 70.87 | 0.95 | 23.62 | <LOD | <LOD | 2.40 | 10.76 | 136.85 |  |
|  | 19-64 years | 63.04 | 0.60 | 26.75 | <LOD | <LOD | 1.78 | 8.84 | 74.73 |  |
|  | ≥ 65 years | 50.00 | 0.23 | 26.11 | <LOD | <LOD | 0.11 | 6.04 | 116.05 |  |
| BPA | total | 80.74 | 0.84 | 4.64 | <LOD | 0.40 | 1.13 | 2.44 | 48.92 | <0.001** |
|  | 6-11 years | 94.18 | 1.61 | 3.19 | <LOD | 1.01 | 1.92 | 3.75 | 22.26 |  |
|  | 12-18 years | 87.39 | 1.10 | 4.15 | <LOD | 0.57 | 1.38 | 2.88 | 33.21 |  |
|  | 19-64 years | 67.39 | 0.47 | 4.87 | <LOD | <LOD | 0.75 | 1.61 | 44.43 |  |
|  | ≥ 65 years | 65.33 | 0.41 | 4.94 | <LOD | <LOD | 0.55 | 1.45 | 48.92 |  |

GM, Geometric mean; GSD, Geometric standard deviation; SD, Standard deviation; P25, P50, P75, percentile;

LOD, Limit of detection; LOD for MP was 0.03 ng/mL, EP and PP were 0.02 ng/mL, and BPA was 0.11 ng/mL. *p<0.05, **p<0.01.

**Table S3**. 24-hour dietary recall data (cooked weight, g) of the study population

|  | 6-11 years  N=188 ^a^ | | | | 12-18 years  N=228 ^b^ | | | | 19-64 years  N=136 ^a^ | | | | ≥65 years  N=150 | | | |
| --- | --- | --- | --- | --- | --- | --- | --- | --- | --- | --- | --- | --- | --- | --- | --- | --- |
| Food group | Mean | SD | Median | Max | Mean | SD | Median | Max | Mean | SD | Median | Max | Mean | SD | Median | Max |
| Grains | 354.46 | 206.11 | 314.45 | 1059 | 409.20 | 278.35 | 343.58 | 2535 | 483.75 | 307.45 | 412.24 | 2026 | 645.64 | 381.85 | 562.38 | 2623 |
| Rice and their products | 272.30 | 188.75 | 255.34 | 1050 | 280.04 | 196.50 | 232.08 | 935 | 356.75 | 256.82 | 297.95 | 1302 | 501.78 | 346.08 | 427.31 | 2568 |
| Wheat and flour products | 42.67 | 73.71 | 0.00 | 425 | 87.04 | 149.69 | 35.38 | 1405 | 92.11 | 135.59 | 55.05 | 927 | 111.18 | 190.53 | 16.98 | 1206 |
| Starchy roots and tubers and their products | 32.37 | 77.13 | 5.39 | 676 | 34.59 | 58.27 | 12.49 | 513 | 31.35 | 85.13 | 0.00 | 621 | 21.70 | 69.07 | 0.00 | 718 |
| Seeds and their products | 7.12 | 37.84 | 0.00 | 406 | 7.53 | 27.48 | 0.00 | 208 | 3.54 | 15.83 | 0.00 | 146 | 10.98 | 53.30 | 0.00 | 424 |
| Oils | 17.82 | 19.68 | 12.28 | 161 | 19.64 | 20.76 | 14.74 | 197 | 22.24 | 26.73 | 13.88 | 132 | 14.84 | 20.95 | 8.40 | 154 |
| Vegetable oil | 15.94 | 18.14 | 10.36 | 143 | 16.53 | 15.79 | 12.81 | 80 | 17.89 | 23.22 | 11.19 | 132 | 10.06 | 11.38 | 6.97 | 71 |
| Animal fat and oil | 0.93 | 4.73 | 0.00 | 56 | 2.14 | 11.90 | 0.00 | 172 | 0.57 | 1.78 | 0.00 | 11 | 0.81 | 3.48 | 0.00 | 32 |
| Nuts and their products | 0.95 | 4.38 | 0.00 | 30 | 0.96 | 4.63 | 0.00 | 36 | 3.79 | 12.74 | 0.00 | 105 | 3.96 | 17.19 | 0.00 | 143 |
| Poultry | 28.91 | 49.64 | 0.00 | 281 | 51.98 | 79.56 | 9.48 | 408 | 17.05 | 33.05 | 0.00 | 204 | 8.55 | 23.67 | 0.00 | 188 |
| Chicken and their products | 26.37 | 45.93 | 0.00 | 260 | 49.35 | 77.27 | 7.32 | 408 | 13.83 | 30.91 | 0.00 | 204 | 8.11 | 23.63 | 0.00 | 188 |
| Ducks and their products | 1.52 | 9.17 | 0.00 | 77 | 2.42 | 12.84 | 0.00 | 125 | 3.23 | 15.06 | 0.00 | 104 | 0.34 | 2.51 | 0.00 | 25 |
| Other poultry and their products | 1.03 | 8.01 | 0.00 | 89 | 0.21 | 3.15 | 0.00 | 48 | 0.00 | 0.00 | 0.00 | 0 | 0.11 | 1.30 | 0.00 | 16 |
| Livestock | 69.85 | 79.43 | 45.88 | 434 | 82.27 | 81.70 | 61.52 | 365 | 76.72 | 84.12 | 56.95 | 487 | 60.29 | 78.07 | 39.02 | 455 |
| Pork and their products | 65.16 | 74.15 | 44.17 | 429 | 71.83 | 70.92 | 55.23 | 361 | 70.08 | 83.98 | 45.41 | 487 | 53.16 | 70.10 | 35.61 | 455 |
| Beef and their products | 4.05 | 21.77 | 0.00 | 206 | 9.24 | 34.89 | 0.00 | 269 | 3.41 | 18.54 | 0.00 | 174 | 2.75 | 16.83 | 0.00 | 153 |
| Other livestock and their products | 0.64 | 7.91 | 0.00 | 108 | 1.20 | 14.87 | 0.00 | 221 | 3.22 | 15.90 | 0.00 | 103 | 4.38 | 31.18 | 0.00 | 311 |
| Fish and Seafood | 33.21 | 49.56 | 11.31 | 269 | 41.92 | 60.56 | 16.79 | 355 | 62.41 | 90.53 | 30.84 | 554 | 33.68 | 53.24 | 12.14 | 265 |
| Freshwater fishes | 4.34 | 19.90 | 0.00 | 201 | 4.92 | 17.26 | 0.00 | 123 | 9.05 | 27.31 | 0.00 | 164 | 4.02 | 13.10 | 0.00 | 78 |
| Saltwater fishes | 10.85 | 26.16 | 0.00 | 220 | 8.87 | 26.72 | 0.00 | 217 | 22.53 | 49.46 | 0.00 | 391 | 19.26 | 41.18 | 0.00 | 265 |
| Other fishes, viscera, and their products | 7.15 | 19.61 | 0.00 | 122 | 15.96 | 36.42 | 0.00 | 280 | 13.83 | 46.47 | 0.00 | 396 | 6.71 | 22.17 | 0.00 | 176 |
| Other seafood and their products | 10.87 | 30.28 | 0.00 | 269 | 12.18 | 35.06 | 0.00 | 242 | 17.01 | 52.89 | 0.00 | 452 | 3.69 | 15.38 | 0.00 | 121 |
| Protein | 231.57 | 230.50 | 177.11 | 1643 | 218.67 | 230.88 | 154.23 | 1323 | 196.90 | 262.53 | 98.19 | 1776 | 110.18 | 160.44 | 45.83 | 1090 |
| Eggs and their products | 48.69 | 55.32 | 32.37 | 266 | 51.34 | 63.04 | 38.40 | 494 | 30.74 | 40.06 | 12.60 | 187 | 16.36 | 30.24 | 0.00 | 133 |
| Dairy | 116.43 | 157.52 | 0.00 | 663 | 91.91 | 173.39 | 0.00 | 1094 | 48.70 | 103.50 | 0.00 | 499 | 40.71 | 118.18 | 0.00 | 1090 |
| Soybeans and their products | 66.45 | 143.40 | 0.00 | 883 | 75.42 | 150.22 | 0.66 | 1323 | 117.46 | 236.74 | 15.38 | 1776 | 52.38 | 112.64 | 0.00 | 550 |
| Special nutritional food | 0.00 | 0.00 | 0.00 | 0 | 0.00 | 0.00 | 0.00 | 0 | 0.00 | 0.00 | 0.00 | 0 | 0.73 | 4.52 | 0.00 | 36 |
| Vegetables | 174.52 | 152.83 | 132.22 | 1306 | 180.47 | 136.81 | 145.75 | 644 | 293.97 | 249.36 | 227.34 | 1493 | 325.66 | 264.44 | 276.56 | 1746 |
| Dark green leafy vegetables | 77.44 | 89.54 | 50.56 | 528 | 74.87 | 80.96 | 55.77 | 548 | 139.33 | 164.81 | 91.62 | 996 | 145.16 | 171.89 | 110.62 | 1246 |
| Light green leafy vegetables | 55.39 | 80.30 | 25.93 | 499 | 69.63 | 84.01 | 40.25 | 483 | 80.37 | 101.09 | 38.17 | 398 | 95.91 | 140.01 | 38.68 | 858 |
| Bamboo shoots | 4.45 | 14.21 | 0.00 | 105 | 4.88 | 14.26 | 0.00 | 123 | 9.58 | 23.53 | 0.00 | 157 | 9.90 | 23.12 | 0.00 | 113 |
| Cucurbits | 16.26 | 36.00 | 0.00 | 243 | 10.35 | 31.66 | 0.00 | 268 | 42.10 | 120.76 | 0.00 | 839 | 50.20 | 112.91 | 0.00 | 735 |
| Legumes | 2.56 | 7.63 | 0.00 | 42 | 3.05 | 9.75 | 0.00 | 71 | 3.24 | 12.37 | 0.00 | 90 | 3.13 | 14.10 | 0.00 | 108 |
| Mushrooms | 9.84 | 33.52 | 0.00 | 313 | 8.48 | 22.76 | 0.00 | 204 | 9.73 | 27.75 | 0.00 | 195 | 10.37 | 33.26 | 0.00 | 225 |
| Other vegetables and their products | 0.00 | 0.00 | 0.00 | 0 | 0.00 | 0.00 | 0.00 | 0 | 0.00 | 0.00 | 0.00 | 0 | 0.00 | 0.00 | 0.00 | 0 |
| Pickled vegetables | 4.64 | 14.86 | 0.00 | 124 | 5.62 | 15.27 | 0.00 | 92 | 6.01 | 14.82 | 0.00 | 88 | 8.98 | 36.13 | 0.00 | 371 |
| Marine vegetables | 3.94 | 10.62 | 0.00 | 64 | 3.60 | 12.07 | 0.00 | 83 | 3.62 | 14.58 | 0.00 | 143 | 2.02 | 10.69 | 0.00 | 102 |
| Seasoned vegetables | 0.00 | 0.00 | 0.00 | 0 | 0.00 | 0.00 | 0.00 | 0 | 0.00 | 0.00 | 0.00 | 0 | 0.00 | 0.00 | 0.00 | 0 |
| Fruits | 121.60 | 147.90 | 84.86 | 734 | 108.22 | 151.04 | 23.25 | 832 | 252.70 | 259.83 | 182.42 | 1146 | 171.86 | 194.54 | 120.23 | 954 |
| Fresh fruits | 111.20 | 135.59 | 78.46 | 669 | 105.49 | 148.45 | 14.87 | 832 | 240.61 | 251.61 | 170.93 | 1112 | 170.38 | 195.05 | 118.30 | 954 |
| Fruit products | 2.24 | 19.57 | 0.00 | 260 | 1.43 | 7.54 | 0.00 | 64 | 1.19 | 5.73 | 0.00 | 46 | 0.34 | 1.65 | 0.00 | 12 |
| Fruit juice | 8.15 | 41.76 | 0.00 | 319 | 1.31 | 18.72 | 0.00 | 282 | 10.90 | 77.26 | 0.00 | 692 | 1.14 | 14.00 | 0.00 | 172 |
| Snacks | 367.36 | 394.63 | 238.29 | 2443 | 671.28 | 604.33 | 528.93 | 3768 | 742.36 | 840.68 | 433.77 | 5261 | 493.32 | 745.35 | 284.43 | 5381 |
| Bread | 38.94 | 56.26 | 0.00 | 263 | 59.01 | 82.19 | 29.76 | 557 | 17.14 | 37.11 | 0.00 | 206 | 7.03 | 25.11 | 0.00 | 157 |
| Pastries and cookies | 33.00 | 69.19 | 0.00 | 537 | 26.46 | 51.68 | 0.00 | 272 | 21.60 | 45.02 | 0.00 | 239 | 16.87 | 66.22 | 0.00 | 488 |
| Candy | 2.86 | 9.77 | 0.00 | 64 | 5.60 | 24.57 | 0.00 | 295 | 2.27 | 12.41 | 0.00 | 122 | 1.14 | 7.99 | 0.00 | 83 |
| Ice products and sweetened beverages | 274.60 | 380.91 | 154.61 | 2273 | 515.80 | 554.13 | 349.42 | 3463 | 669.90 | 833.46 | 372.50 | 5259 | 466.11 | 745.35 | 262.39 | 5381 |
| Processed fruit juice | 10.47 | 54.83 | 0.00 | 379 | 52.27 | 175.96 | 0.00 | 1620 | 19.25 | 89.45 | 0.00 | 649 | 1.87 | 13.51 | 0.00 | 133 |
| Other snacks | 7.49 | 30.76 | 0.00 | 183 | 12.13 | 63.69 | 0.00 | 721 | 12.20 | 67.19 | 0.00 | 516 | 0.29 | 3.54 | 0.00 | 43 |
| Alcohol | 1.12 | 4.04 | 0.00 | 32 | 3.80 | 24.30 | 0.00 | 279 | 25.41 | 124.49 | 0.10 | 1035 | 15.11 | 76.14 | 0.00 | 625 |
| Seasonings | 49.34 | 69.38 | 26.45 | 455 | 52.53 | 60.91 | 30.17 | 380 | 58.79 | 165.23 | 22.92 | 1677 | 27.19 | 36.56 | 15.01 | 285 |
| Sugar | 6.42 | 19.60 | 0.59 | 150 | 6.70 | 18.08 | 0.67 | 204 | 15.42 | 82.99 | 0.12 | 851 | 1.91 | 11.11 | 0.00 | 133 |
| Salt | 1.95 | 2.16 | 1.22 | 14 | 2.59 | 2.58 | 1.82 | 16 | 2.19 | 3.11 | 1.32 | 23 | 2.42 | 3.14 | 1.29 | 18 |
| Soy sauce | 11.90 | 18.86 | 5.21 | 139 | 10.63 | 15.69 | 5.27 | 117 | 11.41 | 17.13 | 5.08 | 122 | 7.71 | 13.71 | 1.31 | 68 |
| Other seasonings | 29.07 | 65.76 | 5.84 | 451 | 32.61 | 55.65 | 10.68 | 356 | 29.77 | 127.95 | 10.73 | 1475 | 15.15 | 29.52 | 6.58 | 266 |
| Others | 312.48 | 328.33 | 230.03 | 2547 | 326.78 | 334.42 | 252.98 | 1812 | 332.43 | 414.47 | 239.22 | 3165 | 276.54 | 532.21 | 196.29 | 6138 |
| Instant noodles | 20.17 | 94.51 | 0.00 | 829 | 5.26 | 33.99 | 0.00 | 373 | 6.81 | 33.38 | 0.00 | 289 | 4.44 | 33.64 | 0.00 | 335 |
| Sandwiches, burgers | 8.71 | 63.78 | 0.00 | 677 | 0.36 | 5.48 | 0.00 | 83 | 5.93 | 38.35 | 0.00 | 357 | 0.00 | 0.00 | 0.00 | 0 |
| Steamed buns, dumplings | 31.58 | 88.68 | 0.00 | 710 | 40.89 | 122.13 | 0.00 | 703 | 44.76 | 109.63 | 0.00 | 533 | 16.66 | 55.18 | 0.00 | 350 |
| Soup | 249.70 | 304.45 | 169.62 | 2547 | 274.53 | 299.18 | 214.04 | 1748 | 270.46 | 368.89 | 192.01 | 2806 | 255.18 | 530.80 | 175.26 | 6138 |
| Others | 2.31 | 12.03 | 0.00 | 123 | 5.74 | 28.66 | 0.00 | 281 | 4.48 | 43.26 | 0.00 | 491 | 0.27 | 3.29 | 0.00 | 40 |

^a^: One missing data; ^b^: Two missing data

**Table S4.** Relationship between urinary parabens and bisphenol A concentrations and 24-hour dietary recall data based on multiple regression analyses.

|  | **6-11 years** (n= 189) ^a^ | | | | **12-18 years** (n= 230) ^a^ | | | **19-64 years** (n= 137) ^a^ | | | **≥65 years** (n= 150) ^a^ | | |
| --- | --- | --- | --- | --- | --- | --- | --- | --- | --- | --- | --- | --- | --- |
|  | β | 95% CI | *p*-value | β | | 95% CI | *p*-value | β | 95% CI | *p*-value | β | 95% CI | *p*-value |
| MP (μg/g creatinine) | | | | | | | | | | | | | |
| Constant | 52.093 | | | 12.868 | | | | -7.068 | | | 42.869 | | |
| Grains | -0.084 | -0.315 – 0.147 | 0.476 | 0.002 | | -0.024 – 0.028 | 0.885 | -0.009 | -0.115 – 0.098 | 0.875 | 0.003 | -0.100 – 0.106 | 0.954 |
| Vegetables | -0.035 | -0.342 – 0.272 | 0.824 | -0.008 | | -0.059 – 0.042 | 0.744 | -0.060 | -0.188 – 0.069 | 0.362 | 0.034 | -0.114 – 0.183 | 0.649 |
| R | 0.095 | | | 0.123 | | | | 0.218 | | | 0.045 | | |
| R^2^ | 0.009 | | | 0.015 | | | | 0.048 | | | 0.002 | | |
| EP (μg/g creatinine) | | | | | | | | | | | | | |
| Constant | -2.181 | | | -1.839 | | | | -13.678 | | | -1.664 | | |
| Oils | -0.038 | -0.198 – 0.121 | 0.638 | -0.006 | | -0.086 – 0.074 | 0.881 | -0.011 | -0.173 – 0.152 | 0.898 | **0.194** | **0.009 – 0.378** | **0.040*** |
| Poultry | 0.038 | -0.023 – 0.100 | 0.216 | -0.016 | | -0.036 – 0.005 | 0.145 | -0.079 | -0.211 – 0.053 | 0.241 | -0.058 | -0.200 – 0.104 | 0.480 |
| Livestock | 0.002 | -0.037 – 0.040 | 0.930 | 0.003 | | -0.018 – 0.024 | 0.792 | 0.018 | -0.038 – 0.074 | 0.531 | -0.014 | -0.064 – 0.036 | 0.581 |
| Fish and seafood | 0.002 | -0.059 – 0.062 | 0.952 | -0.017 | | -0.044 – 0.011 | 0.228 | 0.005 | -0.042 – 0.053 | 0.823 | 0.053 | -0.022 – 0.129 | 0.164 |
| Seasonings | -0.007 | -0.052 – 0.037 | 0.743 | 0.022 | | -0.006 – 0.050 | 0.119 | -0.005 | -0.033 – 0.023 | 0.727 | 0.014 | -0.094 – 0.122 | 0.800 |
| R | 0.135 | | | 0.200 | | | | 0.333 | | | 0.234 | | |
| R^2^ | 0.018 | | | 0.040 | | | | 0.111 | | | 0.055 | | |
| PP (μg/g creatinine) | | | | | | | | | | | | | |
| Constant | 0.086 | | | 4.591 | | | | -13.209 | | | 5.748 | | |
| Grains | -0.006 | -0.017 – 0.005 | 0.284 | -0.003 | | -0.011 – 0.005 | 0.460 | -0.005 | -0.017 – 0.007 | 0.442 | -0.003 | -0.013 – 0.007 | 0.521 |
| Oils | 0.028 | -0.086 – 0.143 | 0.625 | 0.001 | | -0.102 – 0.105 | 0.977 | 0.079 | -0.062 – 0.219 | 0.271 | -0.022 | -0.204 – 0.161 | 0.816 |
| Fruits | 0.013 | -0.002 – 0.027 | 0.089 | -0.004 | | -0.018 – 0.009 | 0.533 | 0.008 | -0.007 – 0.022 | 0.299 | 0.001 | -0.018 – 0.020 | 0.918 |
| Alcohol | 0.023 | -0.523 – 0.570 | 0.933 | -0.029 | | -0.114 – 0.057 | 0.509 | -0.003 | -0.033 – 0.027 | 0.823 | -0.018 | -0.067 – 0.031 | 0.474 |
| Seasonings | 0.012 | -0.020 – 0.044 | 0.475 | -0.011 | | -0.045 – 0.024 | 0.552 | 0.016 | -0.006 – 0.039 | 0.160 | 0.039 | -0.063 – 0.141 | 0.449 |
| R | 0.222 | | | 0.164 | | | | 0.386 | | | 0.135 | | |
| R^2^ | 0.049 | | | 0.027 | | | | 0.149 | | | 0.018 | | |
| BPA (μg/g creatinine) | | | | | | | | | | | | | |
| Constant | 2.292 | | | 0.784 | | | | 1.452 | | | 3.400 | | |
| Alcohol | 0.013 | -0.121 – 0.147 | 0.847 | 0.003 | | -0.013 – 0.019 | 0.708 | -0.001 | -0.003 – 0.002 | 0.668 | -0.004 | -0.015 – 0.007 | 0.528 |
| R | 0.033 | | | 0.095 | | | | 0.042 | | | 0.086 | | |
| R^2^ | 0.001 | | | 0.009 | | | | 0.002 | | | 0.007 | | |

a: Covariates including sex and other food groups. *p<0.05.

**Table S5.** Distributions of parabens and bisphenol A hazard quotients in different age groups

|  | **Group** | **n** | **Min** | **P25** | **P50** | **P75** | **Max** | **p-value ^a^** |
| --- | --- | --- | --- | --- | --- | --- | --- | --- |
| **Methyl Paraben** | 6-11years | 189 | 1.98E-07 | 0.001 | 0.002 | 0.003 | 0.04 | 0.002** |
|  | 12-18years | 229 | 1.02E-07 | 0.001 | 0.001 | 0.004 | 0.005 |  |
|  | 19-64years | 136 | 2.64E-07 | 0.0001 | 0.0002 | 0.0006 | 0.01 |  |
|  | ≥ 65 years | 147 | 1.01E-07 | 0.0001 | 0.0001 | 0.0003 | 0.01 |  |
|  | Total | 701 | 1.01E-07 | 0.001 | 0.002 | 0.004 | 0.04 |  |
| **Ethyl Paraben** | 6-11years | 189 | 7.99E-08 | 5.00E-06 | 1.30E-05 | 2.60E-05 | 3.65E-03 | <0.001** |
|  | 12-18years | 229 | 2.60E-08 | 1.16E-07 | 5.00E-06 | 2.3E-05 | 0.002 |  |
|  | 19-64years | 136 | 6.90E-08 | 8.00E-06 | 2.00E-05 | 0.0001 | 0.001 |  |
|  | ≥ 65 years | 147 | 6.16E-08 | 2.00E-06 | 1.1E-05 | 3.30E-05 | 8.52E-04 |  |
|  | Total | 701 | 2.60E-08 | 2.63E-07 | 1.20E-05 | 3.00E-05 | 3.64E-03 |  |
| **Propyl Paraben** | 6-11years | 189 | 6.63E-07 | 4.00E-06 | 0.0003 | 0.0009 | 0.02 | 0.003** |
|  | 12-18years | 229 | 3.31E-07 | 3.00E-06 | 0.0003 | 0.0014 | 0.03 |  |
|  | 19-64years | 136 | 7.15E-07 | 2.00E-06 | 0.0004 | 0.0016 | 0.02 |  |
|  | ≥ 65 years | 147 | 4.26E-07 | 2.00E-06 | 5.00E-06 | 0.0010 | 0.02 |  |
|  | Total | 701 | 3.31E-07 | 3.00E-06 | 0.0003 | 0.0013 | 0.03 |  |
| **Bisphenol A** | 6-11years | 189 | 4.52 | 111.3 | 171.6 | 261.0 | 3201 | <0.001** |
|  | 12-18years | 229 | 1.58 | 46.59 | 100.8 | 167.7 | 3434 |  |
|  | 19-64years | 136 | 2.43 | 10.20 | 68.51 | 149.53 | 1707 |  |
|  | ≥ 65 years | 147 | 2.38 | 8.83 | 38.29 | 108.76 | 3193 |  |
|  | Total | 701 | 1.58 | 32.8 | 100.8 | 191.1 | 3434 |  |

^a^ Comparison of different age groups by Kruskal-Wallis test. **p<0.01.

**Table S6.** Urinary concentrations of parabens in the general population of various countries

| Author, study, year, country | Results units | Population age (N) | MP | | | | EP | | | | PP | | | |
| --- | --- | --- | --- | --- | --- | --- | --- | --- | --- | --- | --- | --- | --- | --- |
|  |  |  | DR% | GM | P50 | P95 | DR% | GM | P50 | P95 | DR% | GM | P50 | P95 |
| This study, Taiwan | ng/mL | Total (706) | 98.3 | 14.86 | 16.91 | 254.48 | 71.2 | 0.43 | 1.06 | 17.19 | 65.4 | 0.59 | 1.76 | 40.81 |
|  |  | 6-11 years (189) | 98.4 | 12.59 | 13.98 | 224.59 | 77.7 | 0.49 | 1.06 | 9.43 | 72.4 | 0.70 | 1.69 | 31.61 |
|  |  | 12-18 years (230) | 98.7 | 16.21 | 19.32 | 168.08 | 54.3 | 0.19 | 0.74 | 17.17 | 70.8 | 0.95 | 2.40 | 55.73 |
|  |  | 19-64 years (137) | 97.1 | 19.70 | 20.97 | 472.02 | 85.5 | 1.14 | 1.31 | 45.39 | 63.0 | 0.60 | 1.78 | 41.34 |
|  |  | Over 65 years (150) | 98.0 | 12.40 | 14.26 | 96.96 | 75.3 | 0.50 | 1.02 | 13.59 | 50.0 | 0.23 | 0.11 | 28.96 |
|  | μg/g-cre. | Total (706) | 98.3 | 15.69 | 17.54 | 208 | 71.2 | 0.45 | 0.97 | 18.7 | 65.4 | 0.62 | 1.60 | 42.5 |
|  |  | 6-11 years (189) | 98.4 | 13.69 | 14.23 | 129.08 | 77.7 | 0.53 | 0.97 | 8.96 | 72.4 | 0.77 | 1.59 | 29.46 |
|  |  | 12-18 years (230) | 98.7 | 12.43 | 13.94 | 104.78 | 54.3 | 0.15 | 0.37 | 16.36 | 70.8 | 0.73 | 2.00 | 33.06 |
|  |  | 19-64 years (137) | 97.1 | 22.32 | 23.64 | 392.12 | 85.5 | 1.29 | 1.63 | 65.96 | 63.0 | 0.68 | 2.31 | 47.35 |
|  |  | Over 65 years (150) | 98.0 | 19.26 | 21.10 | 161.21 | 75.3 | 0.78 | 1.50 | 26.72 | 50.0 | 0.35 | 0.18 | 60.79 |
| NHANES, 2010, USA | μg/g-cre. | Total (2548) | 99.1 | 55.0 | 58.8 | 902 | 42.4 | - | <LOD | 66.5 | 92.7 | 7.71 | 8.27 | 263 |
|  |  | 6-11 years (356) |  | 36.8 | 26.9 | 1540 |  | - | <LOD | 13.4 |  | 3.75 | 2.70 | 125 |
|  |  | 12-19 years (702) |  | 40.1 | 41.7 | 549 |  | - | <LOD | 32.0 |  | 6.08 | 5.44 | 175 |
|  |  | 20-59 years (1040) |  | 58.6 | 65.9 | 910 |  | - | <LOD | 70.0 |  | 8.63 | 9.79 | 265 |
|  |  | Over 60 years (450) |  | 67.5 | 86.4 | 988 |  | - | <LOD | 83.4 |  | 8.92 | 12.9 | 278 |
| NHANES, 2018, USA | μg/g-cre. | 6-19 years (1324) | 99.4 | 5.2 | 50.1 |  | 39.2 | n ^a^ | <LOD |  | 95.4 | 2.1 | 5.2 |  |
|  |  | Over 20 years (4730) | 99.6 | 5.9 | 74.6 |  | 51.9 | n ^a^ | 1.6 |  | 95.0 | 2.4 | 8.8 |  |
| CHMS, 2012-2013, Canada | ng/mL | Total (2339) | 91.0 | 21 | 19 | 470 | 41.9 | - | <LOD | - | 79.2 | 2.9 | 2.4 | 110 |
|  |  | 6-11 years (481) | 87.9 | 7.7 | 6.0 | 80 | 20.5 | - | <LOD | 6.8 | 71.7 | 0.99 | 0.71 | 18 |
|  |  | 12-19 years (469) | 93.7 | 15 | 10 | - | 29.8 | - | <LOD | 20 | 82.3 | 2.5 | 1.4 | 250 |
|  |  | 20-39 years (328) | 91.3 | 21 | 21 | - | 44.6 | - | <LOD | 40 | 84.9 | 3.9 | 2.7 | 260 |
|  |  | 40-59 years (284) | 90.3 | 25 | 26 | 430 | 46.4 | - | <LOD | 81 | 76.6 | 2.8 | 2.5 | 100 |
|  |  | 60-79 years (314) | 91.6 | 25 | 30 | 460 | 46.5 | - | <LOD | 73 | 78.5 | 3.7 | 3.2 | 110 |
|  | μg/g-cre. | Total (2338) | 91.0 | 21 | 23 | 620 | 41.9 | - | LOD | 72 | 79.2 | 3.0 | 2.1 | 130 |
|  |  | 6-11 years (481) | 87.9 | 9.8 | 7.5 | 250 | 20.5 | - | <LOD | 6.3 | 71.7 | 1.3 | 0.87 | 22 |
|  |  | 12-19 years (469) | 93.7 | 11 | 9.7 | - | 29.8 | - | <LOD | 23 | 82.3 | 1.9 | 1.1 | 140 |
|  |  | 20-39 years (328) | 91.3 | 17 | 18 | 630 | 44.6 | - | <LOD | 54 | 84.9 | 3.1 | 1.8 | 150 |
|  |  | 40-59 years (284) | 90.3 | 29 | 34 | 610 | 46.4 | - | <LOD | 110 | 76.6 | 3.3 | 2.6 | 120 |
|  |  | 60-79 years (314) | 91.6 | 28 | 36 | 710 | 46.5 | - | <LOD | 80 | 78.5 | 4.3 | 5.4 | 130 |
| Kang et al., 2016, Korea | ng/mL | Total (2541) | 97.7 | 116 | 166 | 1145 | 97.2 | 24.7 | 32.8 | 467 | 96.7 | 11.0 | 15.5 | 300 |
|  |  | 3-12 years (659) |  | 56.2 | 71.6 |  |  | 74.3 | 8.82 |  |  | 5.00 | 3.61 |  |
|  |  | 13-18 years (359) |  | 86.8 | 135 |  |  | 13.7 | 21.7 |  |  | 5.80 | 6.54 |  |
|  |  | 19-69 years (1523) |  | 165 | 254 |  |  | 46.0 | 53.4 |  |  | 17.7 | 24.0 |  |
| GerES V, 2021, Germany | μg/g-cre. | Total (516) | 97 | 6.71 | 4.47 | 325 | 69 | 0.82 | 0.64 | 7.66 | 31 | 0.49 | 0.30^b^ | 7.66 |
|  |  | 3-5 years (99) | 96 | 15.12 | 6.94 | 1130 | 58 | 0.93 | 0.99 | 3.53 | 27 | 0.69 | 0.43 ^b^ | 101 |
|  |  | 6-10 years (166) | 95 | 7.26 | 4.26 | 715 | 71 | 0.92 | 0.72 | 6.54 | 33 | 0.55 | 0.33 ^b^ | 40.7 |
|  |  | 11-13 years (103) | 99 | 3.99 | 3.13 | 71.3 | 65 | 0.53 | 0.46 | 7.36 | 24 | 0.32 ^b^ | 0.21 ^b^ | 3.20 |
|  |  | 14-17 years (149) | 99 | 5.21 | 4.46 | 261 | 76 | 0.91 | 0.60 | 10.1 | 36 | 0.47 | 0.21^b^ | 10.0 |
| Huang et al., 2022, Taiwan | ng/mL | 7-17 years (95) | 100 | 65.7 | 149 | 826 | 68.4 | 2.60 | 13.3 | 95.5 | 77.9 | 7.91 | 57.4 | 201 |
|  |  | 18-97 years (271) | 100 | 382 | 397 | 1024 | 100 | 39.6 | 38.8 | 107 | 100 | 109 | 117 | 225 |

^a^: Select summary statistics are not reported for ethyl paraben (EP) given their low detection frequencies; these statistics are denoted as “n ^a^”.

^b^: corresponding volume-based value is <LOQ.

- not calculated: proportion of results below the LOD was too high to provide a valid result.

**Table S7.** Urinary concentrations of bisphenol A in the general population of various countries

| Author, study, year, country | Population age (N) | DR% | BPA (ng/mL) | | | BPA (μg/g-cre.) | | |
| --- | --- | --- | --- | --- | --- | --- | --- | --- |
|  |  |  | GM | P50 | P95 | GM | P50 | P95 |
| This study, Taiwan | Total (706) | 80.7 | 0.84 | 1.13 | 6.63 | 0.88 | 1.19 | 5.43 |
|  | 6-11 years (189) | 94.1 | 1.61 | 1.92 | 6.77 | 1.75 | 2.05 | 6.72 |
|  | 12-18 years (230) | 87.3 | 1.10 | 1.38 | 8.28 | 0.84 | 1.12 | 3.92 |
|  | 19-64 years (137) | 67.3 | 0.47 | 0.75 | 3.55 | 0.53 | 0.71 | 3.73 |
|  | Over 65 years (150) | 65.3 | 0.41 | 0.55 | 5.13 | 0.63 | 0.68 | 8.56 |
| Pirard, C., et al., 2012, Belgium | Total (131) | 74.6 | 2.55 | 2.46 |  | 2.54 | 2.25 |  |
|  | 0-6 years (21) | 100 | 2.71 | 2.65 |  | 3.63 | 3.70 |  |
|  | 7-11 years (21) | 100 | 3.27 | 2.82 |  | 3.04 | 2.90 |  |
|  | 12-19 years (22) | 100 | 2.61 | 2.86 |  | 2.96 | 1.73 |  |
|  | 20-39 years (22) | 95.5 | 2.52 | 2.54 |  | 1.93 | 1.87 |  |
|  | 40-59 years (23) | 95.5 | 2.82 | 2.52 |  | 2.81 | 2.61 |  |
|  | Over 60 years (22) | 95.8 | 2.21 | 1.97 |  | 2.83 | 2.73 |  |
| He, Y., et al., 2009, China | Total (922) | 50 | 0.87 | <LOD |  | 0.38 | <LOD |  |
|  | Under 20 years (65) | 57 | 0.78 | 0.70 |  | 0.92 | 0.60 |  |
|  | 21-30 years (222) | 55 | 0.96 | 0.42 |  | 0.53 | 0.94 |  |
|  | 31-40 years (429) | 54 | 1.06 | 0.57 |  | 0.47 | 0.69 |  |
|  | 41-50 years (160) | 39 | 0.64 | <LOD |  | 0.24 | <LOD |  |
|  | Over 50 years (46) | 15 | 0.26 | <LOD |  | 0.06 | <LOD |  |
| Berman, T., et al., 2014, Israel | 20-74 years (247) | 100 | 2.39 | 2.99 | 18.78 | 1.90 | 2.26 | 14.61 |
| Park, C., et al., 2016, KoNEHS, Korea | Over 19 years (6266) | 78.3 | 0.75 | 0.79 | 5.87 |  |  |  |
| Chang, F.-K.,et al., 2017, Taiwan | Total (123) | 49.6 | 5.81 |  |  |  |  |  |
|  | 11-13 years (52) | 48.1 | 5.61 |  |  |  |  |  |
|  | 19-21 years (71) | 50.7 | 5.95 |  |  |  |  |  |
| NHANES, 2011-2012 | Total (2489) |  | 1.51 | 1.40 | 9.40 | 1.72 | 1.58 | 8.24 |
|  | 6-11 years (396) |  | 1.58 | 1.50 | 8.70 | 2.27 | 2.00 | 14.0 |
|  | 12-19 years (388) |  | 1.69 | 1.70 | 10.0 | 1.64 | 1.48 | 8.74 |
|  | Over 20 years (1705) |  | 1.48 | 1.40 | 9.30 | 1.68 | 1.55 | 7.73 |
| NHANES, 2013-2014 | Total (2686) |  | 1.28 | 1.30 | 7.70 | 1.28 | 1.21 | 5.09 |
|  | 6-11 years (409) |  | 1.43 | 1.40 | 8.00 | 1.81 | 1.80 | 7.03 |
|  | 12-19 years (462) |  | 1.28 | 1.20 | 6.80 | 1.04 | 0.932 | 3.99 |
|  | Over 20 years (1815) |  | 1.26 | 1.30 | 7.80 | 1.27 | 1.21 | 5.09 |
| NHANES, 2015-16 | Total (2651) |  | 1.10 | 1.10 | 6.80 | 1.12 | 1.03 | 5.30 |
|  | 3-5 years (141) |  | 1.21 | 1.30 | 7.40 | 2.86 | 2.73 | 12.5 |
|  | 6-11 years (415) |  | 1.14 | 1.20 | 6.20 | 1.47 | 1.35 | 6.20 |
|  | 12-19 years (405) |  | 1.21 | 1.30 | 7.10 | 0.94 | 0.872 | 4.62 |
|  | Over 20 years (1690) |  | 1.08 | 1.10 | 6.80 | 1.1 | 1.02 | 5.24 |
| CHMS, 2007-2009, Canada | 6-11 years (1031) | 93.5 | 1.3 | 1.3 | 7.1 | 2.0 | 2.0 | 9.8 |
|  | 12-19 years (980) | 93.7 | 1.5 | 1.6 | 8.3 | 1.3 | 1.2 | 6.4 |
|  | 20-39 years (1165) | 92.1 | 1.3 | 1.4 | 7.3 | 1.5 | 1.4 | 6.8 |
|  | 40-59 years (1219) | 87.5 | 1.0 | 1.2 | 6.6 | 1.3 | 1.2 | 7.5 |
|  | 60-79 years (1081) | 88.1 | 0.90 | 0.99 | 5.2 | 1.2 | 1.1 | 7.6 |
| CHMS, 2009-2011, Canada | Total (2560) | 93.8 | 1.2 | 1.2 | 6.7 | 1.2 | 1.0 | 6.9 |
|  | 6-11 years (516) | 93.4 | 1.4 | 1.3 | 9.3 | 1.5 | 1.4 | 10 |
|  | 12-19 years (512) | 94.4 | 1.3 | 1.3 | 7.6 | 1.0 | 0.94 | 5.0 |
|  | 20-39 years (357) | 96.1 | 1.3 | 1.3 | 5.6 | 1.1 | 0.99 | 4.5 |
|  | 40-59 years (360) | 92.7 | 1.2 | 1.2 | 6.7 | 1.2 | 1.1 | 6.9 |
|  | 60-79 years (291) | 91.9 | 1.0 | 0.99 | 6.3 | 1.2 | 1.0 | 6.8 |
| CHMS, 2012-2013, Canada | Total (5670) | 91.7 | 1.1 | 1.1 | 6.6 | 1.1 | 0.99 | 5.9 |
|  | 6-11 years (1004) | 95.9 | 1.2 | 1.2 | 5.3 | 1.5 | 1.4 | 5.3 |
|  | 12-19 years (992) | 92.3 | 1.3 | 1.4 | 8.0 | 1.0 | 0.95 | 5.4 |
|  | 20-39 years (1040) | 91.1 | 1.1 | 1.1 | 6.7 | 1.0 | 0.93 | 5.4 |
|  | 40-59 years (1075) | 93.1 | 1.1 | 1.1 | 7.5 | 1.2 | 0.99 | 6.1 |
|  | 60-79 years (1038) | 88.4 | 0.88 | 0.88 | 5.5 | 1.0 | 0.99 | 4.7 |
